# Dialogue and sports supplementation: reflections from the social sciences: a systematic review

**DOI:** 10.3389/fspor.2025.1719247

**Published:** 2026-01-21

**Authors:** Alexis Sossa Rojas, Manuel Zoccola Cisterna

**Affiliations:** 1 Society and Health Research Center, Universidad Mayor, Santiago, Chile; 2 Pontificia Universidad Catolica de Chile, Santiago, Chile

**Keywords:** athletic performance, doping, nutritional supplement, performance enhancement, social sciences

## Abstract

**Introduction:**

This systematic review investigates sports supplementation research from a social science perspective, focusing on the period from 2014 to 2024. Given the absence of a standard definition and shared taxonomy, the article explores the intricate interrelations among nutritional, psychological, commercial, cultural, historical, and sociological dimensions that constitute this field.

**Methods:**

A systematic review methodology was employed in accordance with the PRISMA protocol. Initially, 440 articles were screened, leading to a final selection of 56 relevant papers distributed across five central themes.

**Results:**

The analysis revealed complex interrelations among various dimensions, including nutritional, psychological, commercial, cultural, historical, and sociological factors. The five identified themes are as follows: Attitudes towards Supplements and Doping (*N* = 18): This theme explores psychosocial influences on substance use. Gateway to Doping (*N* = 9): It highlights the progression from initial supplement use to the adoption of prohibited substances. Networks and Key Actors (*N* = 8): This aspect maps the social and professional interactions that influence athletes' decisions regarding supplementation. The Grey Area of Supplementation (*N* = 12): It addresses underrepresented populations and scientific uncertainties surrounding supplementation. Conceptual Ambiguity (*N* = 9): This theme confronts the ongoing lack of universal definitions and classifications in the field.

**Discussion:**

The findings support a dual taxonomy for classifying supplements into functional foods, individual nutrients, ergogenic supplements, and multi-ingredient products. Furthermore, they establish a hierarchy of scientific evidence, positioning randomised controlled trials as the “gold standard.” This research underscores how contemporary diets integrate cultural traditions, scientific advancements, and technological developments, while emphasising critical dualities: health vs. energy metabolism, risk vs. benefit, and safety vs. effectiveness.

## 1 Introduction

Sports supplements represent an intersection of technology, nutrition, and physical activity, where innovation and sports nutrition play fundamental roles in the pursuit of optimal performance (1–3, 79, 81, 87). While these elements are easily identifiable, their interrelationships and effects can be complex and vary significantly across cultural contexts. Furthermore, the lack of a standard definition and shared taxonomy complicates comparisons between regulations in different countries, influencing research, sports practice, and a comprehensive understanding of sports supplementation.

This article reviews the study of supplementation from a social science perspective, focusing on sports supplements and encompassing both recreational and elite physical activity. Sports supplementation is understood as a multifaceted phenomenon encompassing nutritional, psychological, commercial, cultural, historical, sporting, and sociological dimensions, highlighting that current “healthy diets” extend beyond strict biological standards. These diets integrate cultural traditions, scientific advancements, and technological developments. Additionally, this field faces significant challenges in global systems of food production, distribution, and advertising, underscoring the analysis of sports supplementation as a complex and essential task in contemporary society.

Studies on sports supplementation address various dimensions, from attitudes towards its use to the impact of support networks on decision-making regarding supplementation. In the absence of a standard definition, these analyses promote an open and critical dialogue. Although primarily arising from the health sciences, supplements are intrinsically linked to social factors, functioning as a food phenomenon that raises debates over regulations, education, and health. Understanding supplements as an integral part of many contemporary diets is crucial, as they reveal cultural trends, group influences on decision-making, and the complexities associated with their use, including the sometimes-fine line between supplementation and doping.

In recent decades, the use of sports supplements has grown significantly, not only among elite athletes but also in the general population (4, 5). A recurring theme in the literature is the tension between the desire to enhance performance (and aesthetics) and the ethical and health concerns associated with supplement use. This tension manifests in debates over the line that separates legal supplementation from doping, and how this distinction is perceived and negotiated by athletes, coaches, and sports organisations.

Moreover, more recent research has begun to explore how social media and digital platforms influence the dissemination of information about supplements and the formation of attitudes towards their use (6, 7). This aspect is particularly relevant in the digital information age, where athletes have access to vast amounts of information, some of which is not scientifically backed.

The complexity of this phenomenon is reflected in the diversity of methodological approaches employed in its study, ranging from quantitative analyses of usage patterns to in-depth qualitative studies of athletes' and amateur consumers’ experiences and perceptions. This literature review integrates elements of sports nutrition, diet as a food phenomenon, sport as physical activity, and sports supplements as technological products. Through this approach, the main trends in the literature on sports supplementation from the social sciences are identified and analysed, covering the period from 2014 to 2024. Our research question is: How do social science perspectives illuminate the ethical and social implications of performance-enhancing substances in sports?

The reviewed research has been organised into five central themes that reflect key concerns in nutrition from the social sciences: (1) Attitudes and behaviours towards supplements and doping; (2) Networks and Key Actors; (3) Gateway to Doping; (4) Grey Area of Supplementation; and (5) Conceptual Ambiguity.

## 2 Background: sports supplements and doping: context and considerations

¿What is a supplement? Why is it important to study it? Who studies supplementation from a social science perspective, and how has it been done? Is supplementation and doping the same thing? This section sheds light on these essential questions.

Based on our systematic review, we can conclude that there is no standard definition of what constitutes a supplement. All authors face the problem of conceptual ambiguity, proposing their own operational definitions to address this limitation in their respective studies. In the specialised literature, various terms are used to refer to these products, including “Dietary Supplement,” “Nutritional Supplement,” “Sports Supplement,” “Sporting Foods,” and “Ergogenic Aids,” among others. Generally, authors encounter two main tasks: (1) Choosing a legal/institutional reference framework, whether it be the Dietary Supplement Health and Education Act (DSHEA), the Food and Drug Administration (FDA), the International Olympic Committee (IOC), or the Australian Institute of Sport (AIS); and (2) Establishing how they connect these products to the sporting domain.

It is not sufficient to simply use the term “sports supplement.” Some authors classify supplements based on criteria such as form/ingestion (e.g., gels, bars, or drinks) or by nutritional function (e.g., vitamins, proteins, creatine). However, a common element among all these authors is the “reason” for including supplements in athletes' diets: the pursuit of improved sports performance, whether by optimising performance in training or competitions, fulfilling specific nutritional requirements, or facilitating recovery. Regarding the terms, these function as collective products within the sports supplement industry. While their use may be confusing, they can be generally understood as long as they meet the two aforementioned tasks: providing a clear reference framework and establishing a specific link to the sporting domain.

This lack of consensus not only complicates the understanding of what constitutes a sports supplement but can also lead to confusion in its use and regulation. Despite this vagueness, the popularity of supplements has increased significantly, driven by a growing awareness of nutrition and its impact on sports performance (4, 5).

As a technological innovation, biotechnology has positioned supplements as a revolutionary approach to dietary strategies. While their intake may seem merely an individual complement, it necessitates a comprehensive evaluation by the athlete and their environment. In this sense, nutritional supplements directly engage with the material, the individual, the collective, and the global. The term “sports” is included as they form an integral part of the athlete's diet. Functionally, they can be used to meet nutritional requirements, enhance performance, or facilitate recovery. Although their inclusion in the diet should be done under the guidance of health professionals, it is questionable whether this recommendation is always followed.

Historically, athletes' diets have undergone processes of standardisation and homogenization, driven by trends aimed at normalising dietary parameters to maximise performance (8, 9). Physical activity, whether recreational or elite, modifies eating habits and increases energy expenditure, necessitating a greater intake of nutrients. This dietary planning is influenced by factors such as the athlete's morphological characteristics, body composition, and the discipline practised, thereby determining the predominant energy substrates and metabolic pathways. Additionally, short and long-term goals, as well as the athlete's competitive schedule, must be considered (9, 79).

To understand the athlete's diet, it is vital to analyse the binomial of food/energy metabolism. For most of the population, physical activity does not require significant dietary adjustments, as following the basic principles of a balanced diet is sufficient (9, 79). However, for athletes, meeting nutritional needs solely through food may prove inadequate. In response to this challenge, both the athlete and their environment (coaches, trainers, nutritionists) resort to supplementation to achieve nutritional goals that optimise their performance.

Furthermore, many individuals who do not lead strictly athletic lives also consume these products. This can be attributed to various social and cultural reasons. The popularity of a healthy lifestyle, the promotion of physical appearance on social media, and the influence of celebrities and athletes in supplement marketing have made these products appealing to those seeking to improve their health or appearance, even without a rigorous training regimen.

When evaluating the role of supplements in contemporary society, especially for athletes, initial dietary recommendations should focus on planning a varied and balanced diet, both in quantity and quality, to optimise adaptation to training and competition (9, 79, 86). This planning must consider the relationship between energy expenditure, training programmes, and body composition (9, 79). Thus, sports practice introduces a new binomial: health/energy metabolism. Without proper nutrition, achieving maximum performance is impossible; a deficient diet can even compromise the benefits of the best training and negatively impact the athlete's health (9, 79).

Advancements in biotechnology and nanotechnology have propelled the development and commercialisation of sports supplements. Biotechnology focuses on the evaluation and production of high-quality nutritional supplements, innovating in chemical composition and production processes (81). Conversely, nanotechnology optimises the release and absorption of nutrients within the body. However, the decision to use these supplements is not straightforward, as the wide variety in their composition and characteristics complicates their integration into training programmes for athletes across different disciplines (81). Many sports nutrition products combine macro and micronutrients, as well as phytochemicals, which, while individually supported, lack evidence as a complete mixture (81).

A significant concern is the potential presence of undeclared doping substances in supplements, raising a crucial binomial: risk/benefit. Athletes and their support systems must carefully evaluate supplement use, weighing the benefits for training and recovery against the risk of contamination. Violating WADA (World Anti-Doping Agency) regulations can occur not only through the direct consumption of prohibited substances but also through the use of products with inadequate information about their composition. The lack of knowledge regarding ingredients and production processes is a critical factor in this evaluation.

Doping prevention in sports has been established as a pillar of fair competition. In the quest for greater performance, ergogenic aids have emerged as a widespread option among athletes. Among these aids, our interest centres on nutritional ergogenic supplements that may improve capacity, physical performance, or sports performance.

It is essential to clarify that a nutritional supplement is not in itself a form of doping nor should it be automatically classified as a drug, although various researchers have included them within the category of PEDs (Performance-enhancing Drugs) (10–12). Besides, the WADA list of prohibited substances does not directly mention supplements.

Among the substances considered as “drugs” are: anabolic-androgenic steroids (AAS), anabolic-androgenic precursors, selective androgen receptor modulators (SARMs), anti-oestrogens, aromatase inhibitors, human growth hormone (hGH), insulin, amphetamines and derivatives, stimulants (methylphenidate, ephedrine, cocaine, to name a few). It is important to note that caffeine is no longer on the prohibited list but is included in the WADA Monitoring Programme (10–13).

In this context, Martínez-Sanz et al. (14) address doping through supplementation from the perspective of unintentional doping. This concept refers to positive results in anti-doping tests due to the consumption of supplements containing undeclared prohibited substances or quantities that do not correspond to those indicated in accordance with the regulations of organisations such as WADA. Nevertheless, studies analyse supplements as an integral part of athletes' ergogenic aids, emphasising individual responsibility for the presence of prohibited substances in these products. Unlike other approaches, they do not present this type of doping as equivalent to the deliberate use of drugs, but rather as a “safety problem” and a public health concern.

Recently, the evaluation of sports supplements has become more accessible, focusing on the safety/effectiveness dichotomy (2). Six key supplements with solid scientific backing have been identified, with their mechanisms and effects on athletes' bodies progressively documented: beta-alanine, sodium bicarbonate, creatine, caffeine, nitrates, and protein (2, 15). This information is crucial for athletes and their support teams to prepare for potential contingencies by conducting a thorough review of the scientific evidence regarding the various supplements to include in their nutritional plans. Each supplement has its own mechanisms of action, safety profiles, adverse effects, protocols, and dosages. Therefore, increasing awareness among athletes, health professionals, and support teams is essential to promote safe practices and address public health concerns associated with the misuse of supplements (15, 16).

Although supplements are common among athletes (and increasingly among non-athletes), their benefits do not always meet the expectations generated by commercial narratives. Therefore, it is indispensable to prioritise conventional nutrition before resorting to supplementation, always under a dietary plan supervised by health professionals and considering the health/energy metabolism, risk/benefit, and safety/effectiveness binomials. In response to this need, international organisations have developed evaluation tools to guide decision-making regarding the use of supplements.

The IOC statement presents a decision tree for athletes that explores the risk-benefit binomial (15). This model is structured around two fundamental aspects: the potential rewards of using supplements and their associated costs and risks. Regarding rewards, three main objectives have been identified: correcting nutritional deficiencies, achieving specific nutritional goals, and enhancing physiological or biochemical functions that impact performance (15). Regarding costs, three key risks are identified: the use of ineffective supplements, potential adverse health effects, and the risk of violating anti-doping rules (ADRV) (15).

Evaluating the appropriateness of these supplements requires considering the practicality of their use, consulting with the training team and the medical-scientific support network, and conducting a nutritional assessment that justifies their implementation. The model emphasises the athlete's responsibility in acquiring the product, who must verify its provenance and ensure the absence of prohibited substances in the formulation.

The Spanish Society of Sports Medicine has developed a complementary decision diagram that, unlike the IOC model, emphasises the health/energy metabolism binomial (86). Its logical sequence begins by evaluating whether the athlete's diet includes sufficient “real” food and establishes more specific sequential criteria: the adequacy of exercise and nutrition, clarity of objectives, knowledge of adverse effects and drug interactions, existence of scientific evidence, identification of possible prohibited substances, feasibility of use in competitions, certification of provenance, and documentation of prior positive results (86). Meeting all these requirements allows for the consideration of supplement use, always under strict professional supervision, while the failure to meet any criterion contraindicates its use.

## 3 Methodology

To our knowledge, there is currently no systematic review that specifically addresses the state of the art of supplementation from a social sciences perspective. While several notable works on the subject have been identified (17–20, 84), efforts to consolidate these topics of interest to social scientists are lacking. This article does not seek to provide a comprehensive summary of existing knowledge on supplementation; instead, it aims to explore, characterise, group, and highlight priority thematic areas for future research.

This section documents the methodological process of systematic bibliographic search, outlining the search through four steps: (1) Design, (2) Systematic Search, (3) Inclusion/Exclusion Criteria, and (4) Information Extraction. In addition to these stages, any changes or modifications to the search design are documented.

This systematic review aligns with WADA's efforts to develop a holistic perspective on supplementation and doping. Following WADA's investment in social research since 2005 and the implementation of the new World Anti-Doping Code in 2015, studies such as those by Backhouse et al. (21) analyse the role of social sciences in doping research, highlighting their slow but progressive incorporation into this field. The aim is to position the social sciences as a preventive agent, providing tools to examine how and why athletes dope. This reinterpretation not only promotes a holistic understanding of doping but also invites the social sciences to participate actively in the debate.

Therefore, although the search terms did not specifically focus on doping, several studies centred on this topic were included to avoid potential positive bias regarding supplement use among athletic populations. Similar to the IOC or the Spanish Society of Sports Medicine, this review recommends that the implementation of supplement use be guided by decision-making trees and by consultation with the training team and the medical-scientific support network. The temporal scope of our review spans from 2014 to 2024, reflecting WADA's re-evaluation of the role of social sciences, with the expectation of finding a growing trend of social science researchers dedicated to studying doping and supplementation during this period. The review began on June 20, 2024, and the search was completed on February 19, 2025.

### 3.1 Design

To conduct our review, systematic components were integrated into the research design, following the PRISMA (Preferred Reporting Items for Systematic Reviews and Meta-Analyses) guidelines. This includes defining explicit inclusion and exclusion criteria, conducting a comprehensive search across multiple databases, peer-reviewing relevant studies, and systematically extracting data. Furthermore, a specific search string is developed that combines terms related to supplementation, sport, and the social sciences, aiming to maximise both sensitivity and specificity. This design aims to provide a comprehensive synthesis of existing literature, ensuring the rigour and quality of the work through the application of principles and methods from systematic reviews (22).

### 3.2 Systematic search

The foundation of this work was the selection of keywords that formed the “search string,” facilitating standardised navigation through the chosen databases: (1) Web of Science (WoS), (2) Scopus, and (3) PubMed. For the keywords, three distinct groups were identified (1) terms related to supplementation, (2) terms related to sport, and (3) terms related to social sciences. This classification enabled a more refined and targeted search. In our review, we incorporated a variety of academic resources, including scientific articles, book chapters, and grey literature. An example of the search string used in the different databases:

WOS: TS = ((“Nutritional supplement*” OR “dietary supplement*” OR “sports supplement*” OR “ergogenic aid*” OR “performance enhanc*”) AND (athlete* OR sport* OR “physical activity” OR exercis*) AND (sociol* OR psychol* OR anthropol* OR “social science*” OR ethic* OR decision* OR perception* OR attitude* OR “meaning-making” OR framing OR network* OR “self-affirmation”))

PubMED:((“Nutritional supplement"[Title/Abstract] OR “dietary supplement"[Title/Abstract] OR “sports supplement"[Title/Abstract] OR “ergogenic aid"[Title/Abstract] OR “performance enhancement"[Title/Abstract] OR “performance enhancer"[Title/Abstract]) AND (athlete*[Title/Abstract] OR sport*[Title/Abstract] OR “physical activity"[Title/Abstract] OR exercis*[Title/Abstract]) AND (sociolog*[Title/Abstract] OR psycholog*[Title/Abstract] OR anthropolog*[Title/Abstract] OR “social science"[Title/Abstract] OR “social sciences"[Title/Abstract] OR ethic*[Title/Abstract] OR decision*[Title/Abstract] OR perception*[Title/Abstract] OR attitude*[Title/Abstract] OR “meaning-making"[Title/Abstract] OR framing[Title/Abstract] OR network*[Title/Abstract] OR “self-affirmation"[Title/Abstract]))

SCOPUS: TITLE-ABS-KEY((“Nutritional supplement*” OR “dietary supplement*” OR “sports supplement*” OR “ergogenic aid*” OR “performance enhanc*”) AND (athlete* OR sport* OR “physical activity” OR exercis*) AND (sociol* OR psychol* OR anthropol* OR “social science*” OR ethic* OR decision* OR perception* OR attitude* OR “meaning-making” OR framing OR network* OR “self-affirmation”))

The selection of search terms was conducted through an iterative testing process across the different databases. The initial criterion for including or excluding words was based on their quantitative impact on search results. However, this criterion alone may prove insufficient. Search strings must be broad enough to capture all potentially relevant material, thereby avoiding the loss of resources that could be vital to the research.

The search string syntax varies according to the database used. For Web of Science (WoS), the prefix “TS” is employed, which indicates the search topic and includes terms in the title, abstract, and keywords of the indexed record. In PubMed, “Title/Abstract” is used for words contained in the title and abstract. For Scopus, “TITLE-ABS-KEY” is used, which corresponds to title, abstract, author keywords, and index terms. The Boolean operators “AND” and “OR” ensure an exhaustive and precise search in each database. “AND” links ideas and concepts, requiring the presence of all of them in the results. “OR” connects similar words—such as synonyms, acronyms, or variations of the same concept—broadening the search. These operators must be written in uppercase. Truncation (*) allows searching for different derivations of a word. Quotation marks (“…”) search for exact phrases. Parentheses “()” structure the search and combine terms in an organised manner with Boolean operators.

**Figure 1:**
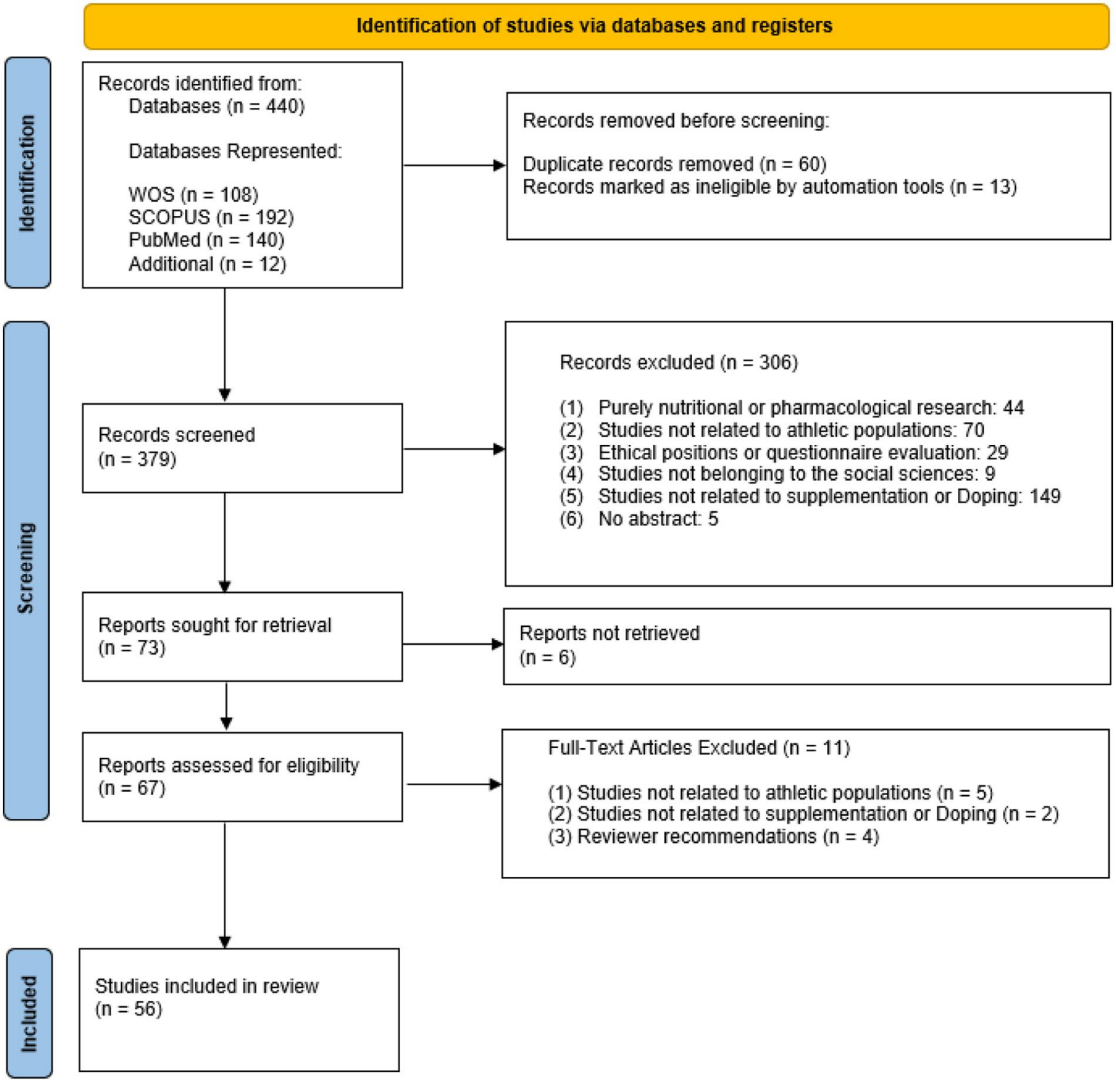
Preferred reporting items for systematic reviews and meta-analyses flow diagram.

Figure 1 illustrates each step of the review procedure, carried out according to the PRISMA guidelines. The systematic review yielded 440 articles. Of these, 73 were eliminated due to duplication or illegibility using automated tools. Additionally, 12 articles were added through manual searches conducted by the researchers. This left 379 articles for review and application of the inclusion and exclusion criteria. After applying the established criteria, 73 articles were selected for full reading. Of these, 6 were excluded because full text was unavailable, resulting in a final count of 56 articles that met all inclusion criteria. These were distributed across five central themes: (1) Attitudes and behaviours towards supplements and doping (*N* = 18); (2) Networks and Key Actors (*N* = 8); (3) Gateway to Doping (*N* = 9); (4) Grey Area of Supplementation (*N* = 12); and (5) Conceptual Ambiguity (*N* = 9).

### 3.3 Inclusion/exclusion criteria

This review established several criteria for article eligibility. Firstly, studies had to be published in English or Spanish in academic journals between the years 2014 and 2024. Furthermore, the research needed to focus on athletes, covering both recreational and elite levels. Notably, there were no restrictions regarding age, gender, dietary practices, or specific sports disciplines, allowing for a comprehensive examination of the topic. This inclusive approach was designed to capture a broad spectrum of perspectives and findings related to supplementation within athletic populations, thereby enriching the overall analysis.

The selected articles were required to concentrate on: studies of perceptions, attitudes, or beliefs regarding sports supplements and doping; research on social or cultural factors influencing the use of supplements or doping substances; analyses of behaviours related to supplementation in athletes; and studies examining decision-making processes concerning the use of supplements or doping substances.

Prior to the review, automated tools were used to identify potential duplicate articles across various databases. We used Rayyan, a platform specialised in literature reviews and systematic reviews, which allows visualisation, duplicate identification, and the application of inclusion/exclusion criteria to our review. The articles were evaluated through title and abstract reviews, applying the established inclusion and exclusion criteria and incorporating articles identified through the authors' manual searches. Both researchers reviewed all abstracts to exclude publications that did not meet the inclusion criteria. To implement blinding, the graduate student first applied the criteria and selected the articles. Subsequently, the principal author reapplied the requirements to that selection. After the selection process, the double-blind procedure was lifted, and the inclusion and exclusion of articles were resolved through deliberation.

Articles were excluded if they: focused solely on the physiological effects of supplements; presented purely nutritional or pharmacological research; were unrelated to athletic populations or supplementation and doping; were written in languages other than Spanish or English; or did not belong to the social sciences. Additionally, complementary refinement criteria were established in the search engines, available as filters within the databases, including a publication-year restriction (2014–2024) and a field restriction to the social sciences.

In the subsequent phase, both authors reviewed the articles and classified them according to different thematic areas. The titles of these areas emerged from the readings themselves, selecting key concepts used transversally across the various articles. For example, in “Attitudes and Beliefs”, both concepts appear as constructs of models that position the social sciences as a preventive agent, providing tools to examine how and why athletes dope. “Gateway to Doping” brings together research based on the hypothesis of the same name. “Networks and Key Actors” includes studies that position the social environment as the primary object of analysis. “Grey Area” explores concerns about the uncertainties surrounding scientific knowledge of supplements. Finally, “Conceptual Ambiguity” refers to the challenge of establishing a standard definition and classification of supplements, a challenge that repeatedly appears in the literature as a pending task.

The selection and naming of these thematic areas were not arbitrary but emerged from an iterative process of identifying recurring concepts and theoretical underpinnings prevalent in the social sciences literature on supplementation. Each theme, therefore, represents a distinct social science lens through which the complex phenomenon of sports supplementation is studied: “Attitudes and Behaviours towards Supplements and Doping” draws heavily on social psychology and theories of reasoned action/planned behaviour; “Networks and Key Actors” aligns with sociological concepts of social networks, organisational behaviour, and community influences; “Gateway to Doping” is rooted in social learning theories and deviance pathways; “Grey Area of Supplementation” reflects critical sociology and science and technology studies debates on knowledge production, regulation, and uncertainty; and “Conceptual Ambiguity” highlights issues of definition and classification, often explored through discourse analysis or sociological studies of knowledge. This approach ensures that our thematic organisation is theoretically robust and facilitates a social science-driven interpretation of the findings.

Once articles were categorised into these themes, a narrative synthesis approach was adopted to integrate the findings comprehensively. This involved systematically aggregating, comparing, and interpreting the results from each study within its assigned theme. We meticulously identified overarching patterns, common threads, and any discrepancies or unique insights that collectively contributed to a comprehensive understanding of each thematic area. This iterative process allowed us to construct coherent thematic narratives that reflect the collective evidence and articulate key arguments, thereby enhancing the analytical depth and transparency of our findings. The goal was not merely to summarise individual studies but to synthesise their contributions into a cohesive understanding of the social science perspective on sports supplementation, framed by our established themes.

### 3.4 Methodological quality assessment

Although it is common in systematic reviews to methodologically evaluate each included article, we have chosen to omit this step in our work, as it is not the primary aim of our review and because no tools are specifically tailored for the social sciences.

Methodological quality is typically assessed using Critical Appraisal Tools (CATs), which help professionals and researchers evaluate the quality and reliability of scientific research. These tools encompass various study designs, quantitative, qualitative, and mixed-methods, with mixed-methods studies being particularly relevant to our systematic review. Mixed-methods appraisal tools have been developed under the premise that they can offer, within a single instrument, criteria for methodological quality applicable to different study designs (23). Among the primary tools are the Mixed Methods Appraisal Tool (MMAT) and the Quality Assessment with Diverse Studies (QuADS).

However, as Rouleau (24) argues, these tools were initially designed for quantitative systematic reviews in health research, meaning that when applied to other fields or research designs, they require adaptation or the development of new tools. Consequently, qualitative and mixed-methods approaches have often been subjected to assumptions and methodological reasoning that fail to account for their unique characteristics, usually defined in terms of their non-numeric nature.

This is evident when consulting materials such as the Dictionary of Epidemiology from the International Epidemiological Association, which defines qualitative research as: “Any type of research that employs nonnumeric information to explore individual or group characteristics, producing findings not arrived at by statistical procedures or other quantitative means” [ (25), p. 223]. However, as Tavory (26) has argued, translating quantitative criteria is a mistake. Currently, the debate surrounding qualitative standards has progressed, leading to numerous proposals for establishing evaluation criteria (27, 28). Nonetheless, it remains unclear, and there is no consensus on which elements make the various methodologies in the social sciences comparable. It is well understood that qualitative research should not conform to quantitative standards; instead, both methodologies should adhere to rigorous and distinct standards.

Consequently, proposing a tool that can standardize methodological evaluation across different designs for application in other disciplines without significant adjustment may be problematic. This is primarily due to the distinctive nature of qualitative methods and their diversity, alongside that of quantitative methods. This is illustrated when examining the information extracted from the tables (see Figures. 1–5), which show the variety of methods and instruments that researchers utilize to develop both statistical and qualitative analytical frameworks.

For example, the MMAT categorizes qualitative studies and describes ethnography as a qualitative approach. However, classifying it in such a manner may compromise its methodological assessment. For anthropology, ethnography is not merely a method; it represents both an object of study and a way of knowing, functioning as a method, theory, and form of literature (29). At the very least, it encompasses a set of methods for approaching what we understand as intellectual viewpoints (ways of thinking or signifying the world) and experiential viewpoints (ways of doing and creating social life) of individuals (30). In the case of quantitative studies, the MMAT distinguishes three designs: randomised controlled trials, non-randomised quantitative studies, and descriptive studies. However, within the social sciences, there exist myriad methodologies, each with its distinct methods, such as Structural Equation Modelling (SEM) or Social Network Analysis (SNA), which utilise subtly different mathematical frameworks compared to those identified in the MMAT.

Although we acknowledge that there are comparable elements across methods and disciplines (31), current tools cannot adequately assess the complexity of social science methods. Therefore, as this work does not aim to evaluate each article methodologically but rather to identify potential thematic areas of interest for the social sciences and establish a starting point for future research, we did not employ a Critical Appraisal Tool. However, we recognize that future studies must address the need to adapt existing tools to the heterogeneity present in the social sciences.

### 3.5 Information extraction

Inspired by previous studies (32, 85), the following information was extracted: Author/Year, Purpose, Participants, Desi, Methods, Sport(s) and Quartile (Tables 1–5).

**Table 1:** Summary of the attitudes towards supplements and doping studies included in the review.

Autor(s)	Purpose	Participants	Desing	Method(s)	Sport(s)	Quartile
Barkoukis et al. (33)	The present study sets out to assess the impact of attributional beliefs about success on the susceptibility for doping use in adolescent athletes.	The sample of the study consisted of 309 adolescent's athletes.	Cross-sectional Quantitative	Questionnaires, variance reduction rate (VRR), linear regression analysis, Mediation effect.	Major teams (i.e., basketball, football, volleyball and handball) and several individual sports (i.e., athletics, swimming, taekwondo, rowing etc.).	Q1
Barkoukis et al. (34)	The present study investigated the effectiveness of a school-based intervention in promoting an anti-doping culture in adolescents.	The sample consisted of 218 high school students, 107 males and 110 females, one student did not report gender.	Experimental Study Quantitative	Questionnaires, intervention and control group, ANOVAs, multilevel analysis, HLM analysis.	Representation of major teams (i.e., basketball, football, volleyball and handball) and several individual sports (i.e., athletics, swimming, Taekwondo, rowing etc.).	Q1
Barkoukis et al. (35)	The present study investigated whether self-affirmation changed exercisers’ intentions to use IPEDs, via the effects of mental construal and message acceptance.	Sixty-eight exercisers who self-reported IPEDs use participated in the study and were randomly assigned to either a self-affirmation or a control group.	Experimental Study Quantitative	Questionnaires, intervention and control group, Pearsons's correlations, T-test, hierarchical regression analysis, Cronbach alpha.	N/A	Q1
Boardley et al. (36)	The current study qualitatively investigated psychosocial processes that support performance enhancing drug use in athletes from a range of sports, using Bandura's social cognitive theory of moral thought and action as the guiding theoretical framework.	The study entailed conducting semi-structured interviews with twelve male athletes	Qualitative	semi-structured interviews, content analysis, operational definitions, coding, indicators of intra- and inter-rater reliability, deductive analysis.	athletics (*n* = 1), swimming (*n* = 2), American football (*n* = 3), boxing (*n* = 1), basketball (*n* = 2), wrestling (*n* = 1), rugby (*n* = 1), and mixed martial arts (MMA; *n* = 1).	Q1
Boardley et al. (37)	To develop Moral Disengagement (MD) and Self-Regulatory Efficacy (SRE) instruments relevant to doping in sport and exercise and provide evidence for the validity and reliability of instrument scores.	Two samples (sample 1 = 318; sample 2 = 300) were utilized in instrument development and score validation and another (sample 3 = 101) in examining test-retest reliability and stability of scores.	Cross-sectional Quantitative	Questionnaires, Satorra–Bentler scaled chi-square, robust comparative fit index, standardized root means square residual, root mean square error of approximation, stability analyses	Participants were team- (e.g., American football, Australian rules football, soccer) or individual- (e.g., swimming, athletics, triathlon) sport or Bodybuilding.	Q1
Campian et al. (38)	This study was to examine the use of PEDs in ultramarathon running and to identify attitudes and beliefs about the usage of PEDs in the sport.	609 ultramarathon runners	Quantitative	Questionnaires, *χ*2 test, independent *t*-test, Cronbach's alpha, Monte Carlo permutation tests.	Ultramarathon running	Q3
Chan et al. (39)	The present investigation aimed to explore beliefs surrounding intentions and motivation to use banned performance-enhancing substances in elite athletes using a qualitative thematic content analysis.	A total of eight teams (total *N* = 57 athletes) from seven different sports.	Qualitative	Questionnaires, focus-group, Interview, thematic content analysis, inductive analysis.	(Track and field, basketball, field hockey, netball, water polo, and swimming)	Q1
Chan et al. (40)	This study examined the modal salient behavioural, normative, and control beliefs within the theory of planned behaviour (TPB) in the context of anti-doping in sport.	410 young athletes	Quantitative	Questionnaires, variance-based structural equation modelling (VB-SEM), factor loadings, cross-loadings, average variance extracted (AVE), composite score reliability, Cronbach's alpha, Goodness of fit (GoF) index, averaged R-squared (ARS), averaged variance inflation factor (AVIF), averaged path coefficient (APC), bootstrapping.	Six individual sports (i.e., athletics track, athletics field, badminton, gymnastics, swimming, and triathlon), and six team sports (i.e., soccer, field hockey, water polo, basketball, rugby, and cricket).	Q1
Christensen et al. (41)	This study examines the culture of DSs from an anti-doping perspective through focus group interviews of male elite ice hockey players with the aim of obtaining a deeper knowledge about the attitudes, motivations, and practices relating to DS use, perceived doping risks, and athleteś sources of inspiration and advice when considering using DSs.	A total of 25 male elite ice hockey.	Qualitative	Semi-Structured Interview, focus group, thematic analysis, data-driven approach, coding, main themes, subthemes, inductive analysis.	Ice Hockey	Q1
Duncan et al. (42)	The purpose of this study was to qualitatively explore the personal and situational factors that contribute to the initiation of doping among adolescent athletes.	The 21 recruited athletes (*n* = 11 females and *n*=10 males)	Social Construction epistemology and a relativist ontology.	face-to-face semi-structured interviews, creative non-fiction storytelling, portraiture, character sketches,	13 different sports including: football (*n*=4), track (*n*=4), water polo (*n*=3), synchronized swimming (*n*=2), volleyball (*n*=2), gymnastics (*n*=2), badminton (*n*=2), baseball (*n*=2), basketball (*n*=1), hockey (*n*=1), soccer (*n*=1), rugby (*n*=1), and wrestling (*n*=1).	
Elbe & Brand (43)	This article examines whether a training program in ethical decision making can change young athletes’ doping attitudes.	69 young elite athletes (34 male, 35 females)	Pre- and post measurement design Quantitative	Random intervention and control group, short interviews, Confirmatory factor analysis, ANOVA, saturated model, questionnaires.	team sports (50.7%; e.g., handball, soccer). Other larger groups of athletes came from track and field (18.8%; e.g., sprint, discus), racquet sports (5.8%; e.g., tennis, golf), or martial arts (4.3%; e.g., judo, karate).	Q1
Kim & Kim (44)	This study aims to evaluate doping knowledge, practices, and attitudes among Korean adult and adolescent elite athletes to provide effective information on anti-doping policies and education programs.	454 Korean elite athletes (249 adults in 23 events and 205 adolescents in 22 events).	Cross-sectional Quantitative	Questionnaires, confirmatory factor analysis, χ2/df RMSEA, TLI, CFI, independent *t*-test, A one-way analysis of variance with a *post hoc* least significance difference (LSD)	athletics, weightlifting, taekwondo, judo and so on, and the endurance category, swimming and cycling; the motor skill category, tennis, fencing, badminton, shooting, golf, etc.; and the team category, handball, rugby, hockey, soccer, volleyball, basketball.	Q2
Jowett et al. (82)	The aim of the present study was to investigate whether perfectionism was related to doping willingness directly, and indirectly via moral disengagement.	204 student athletes (*M* age = 19.12 years, *SD* = 1.17, *n* = 81 females - 39.70%)	Cross Sectional Quantitative	Questionnaire, multiple regression with robust estimators, Comparative Fit Index (CFI), Tucker–Lewis Index (TLI; this is also known as non-normed fit index, NNFI), Standardized Root Mean Square Residual (SRMR), and the Root Mean Square Error of Approximation (RMSEA), bootstrapping.	The athletes competed across different levels of sport, including club (*n* = 131, 64.22%), county (*n* = 18, 8.82%), regional (*n* = 23, 11.28%), national (*n* = 18, 8.82%) and international (*n* = 12, 5.88%).	Q1
Mudrak et al. (45)	In this study, we examine doping among adolescents from a motivational perspective and explore how motivational variables, such as achievement goal orientations and the perceived self-determination of sports activities, may be related to moral attitudes, doping intentions and doping behaviour in adolescents who participate in competitive sports.	The study included 1,035 adolescents participating in competitive sports from all regions of the Czech Republic (mean age = 16.3 years).	Quantitative	Battery of questionnaires, tested hypothesized, structural equation modelling (SEM), chi-square, standardized root mean square residual (SRMR), the root means square error of approximation (RMSEA), the comparative fit index (CFI), correlations.	Competitive sports	Q1
De Oliveira et al. (80)	Was investigated ergogenic aids (EAs) used by Brazilian athletes and their association with performance, sex, sports classification, and modality. It identified the main purposes of EAs and their prescription.	239 athletes (134male and 105 female)	Cross-sectional Quantitative	Questionnaires, Pearson's chi-square test, *post hoc* Goodness-of-fit test, non-centrality parameter *λ*, Phi coefficient and Crame's V, The Shapiro–Wilk test, logistic regression model.	swimming (*n* = 44), track and field (*n* = 32), soccer (*n* = 32), triathlon (*n* = 25), handball (*n* = 19), cycling (*n* = 17), combat sports (*n* = 13), basketball (*n* = 12), trampoline gymnastics (*n* = 11) volleyball (*n* = 9), modern pentathlon (*n* = 9), rugby (*n* = 4), shooting sports (*n* = 4), weightlifting (*n* = 4), and American football (*n* = 4).	Q1
Sadek et al. (46)	Therefore, we aim in this study to assess the knowledge, attitudes, and usage of DSs among Lebanese athletes who practice their sports for at least two years.	455 athletes, 73.1% were males, and 26.9% were females.	Cross-sectional Quantitative	Questionnaires, Chi-square, binary logistic regression, bivariate analysis.	ball games (football, basketball, handball, futsal, beach soccer, tennis, table tennis, squash, and speedball); 27% in combat sports (taekwondo, karate, judo, boxing, wrestling, and mixed martial arts); 16.3% in endurance sports (running, swimming, and cycling); and 13.2% in weightlifting.	Q1
Szűcs and Szakály (18)	We describe the attitudes of different segments of consumers who are engaged in sports on a regular basis towards dietary supplements.	Involving a sample of 737 people (*n*=737).	Quantitative	Questionnaires -based survey, Cramer's association evaluation, Kendall's coefficient of concordance, cross analyses, factor, K-means cluster analysis, discrimination analysis.	The most popular sports among the respondents were as follows: weight training (34.5%), running (29.4%), ball games (18.9%), cycling (15.9%), swimming (9.9%), calisthenics (8.8%), martial arts (6.0%), and aerobics (5.8%). Among ball games, soccer was the most popular, at 8.1%.	Q1
Whitaker et al. (47)	To examine athletes’ implicit and explicit prototype perceptions of performance enhancing substance (PES) users and non-users.	The study involved 226 competitive athletes with a mean age of 27.66 ± 9.74 years.	A cross sectional mixed-method study.	Questionnaires, self-report questions, two Brief Implicit Association Tests online, Dependent *t*-tests, correlations, Spearman's Rank, Two-step cluster analysis, Kruskal Wallis x2, mixed model ANOVA, chi square, log-likelihood.	Participants represented 39 sports with the highest proportions of participants being from cycling, athletics and hockey	Q4
						Q1

**Table 2:** Summary of the getaway to doping studies included in the review.

Autor(s)	Purpose	Participants	Desing	Method(s)	Sport(s)	Quartile
Barkoukis et al. (48)	The aim of the present study was to empirically examine if adolescent athletes who reported NS use displayed more favourable reasoning toward doping use, as compared with their counterparts who do not consume NS.	*N* = From 816 athletes approached, 650 were adolescents (age range between 14 and 20 years old; *M* = 16.09, SD = 1.50). They came from both team and individual sports.	Cross-sectional Quantitative.	Questionnaire, Chi (χ2), One-way analysis of variance and Bonferroni *post-hoc* analysis.	They came from both team and individual sports (football *n* = 156, basketball *n* = 169, volleyball *n* = 115, handball *n* = 34, athletics *n* = 66, swimming *n* = 38, shooting *n* = 1, taekwondo *n* = 4, boxing *n* = 8, water polo *n* = 32, and 27 athletes did not specify their sport).	Q1
Barkoukis et al. (49)	The present study examined if using self-affirmation manipulation, a) changes intentions to use doping and b) influences related social cognitions (i.e., attitudes, social and moral norms, self-efficacy and situational temptation, and anticipated regret) among exercisers who use nutritional supplements, following a brief exposure to doping-related health risk messages.	*N* = Sixty Exercises (43 males) who were currently using nutritional supplements.	Between-group Experimental Quantitative.	Structured Survey, one-way ANOVA, experimental and control groups, Multivariate analysis of variance (MANOVA), Multiple linear regression analysis.	N/A	Q1
García-Grimau (50)	The aim of the present study was to develop an explanatory model of doping susceptibility among competitive track and field athletes using a logistic regression analysis accounting for some morality-related variables which were not explored in previous studies.	*N* = 281 Spanish elite track and field athletes (49.5% women, 48.4% have competed with the national team)	Cross-sectional Quantitative.	Online survey. Logistic regression, Pearson zero-order correlations, conditional regression, Means and/or Factor Analysis.	track and field athletes	Q1
Hurst et al. (51)	The aim of this study was to examine: (a) whether sport supplement use is related to doping 15 and (b) whether sport supplement beliefs mediated this relationship.	*N* = Study 1: Competitive male (*n* = 417) and female (*n* = 191) athletes. Study 2: Four-hundred and eighty-one competitive athletes.	Quantitative	Questionnaire, Pearson zero-order correlations, Bootstrapping, Regression.	N/A	Q1
Hurst et al. (52)	1) examine whether users and non-users of different types of sport supplements vary in doping attitudes and sport supplement beliefs, and 2) determine whether the type of sport supplement is directly and indirectly (via sport supplement beliefs) related to doping attitudes.	*N* = Athletes (*N* = 557; 77% male, mean ± standard deviation; age = 20.8 ± 4.5 years, training = 5.7 ± 4.2 h per week, competing = 11.1 ± 5.2 years). We recruited participants from teams (78%) and individual (22%) sports.	Cross-sectional Survey Quantitative	Questionnaires, multiple imputation model, Levene's test of equality of variance, Cohen's d (d) effect size statistic	We recruited participants from team (78%) and individual (22%) sports, who competed at club (26%), county (37%), national (28%) and international (9%) level.	Q1
Hurst et al. (53)	We examined whether Sport supplement use was indirectly related to doping use via sport supplement beliefs, and whether personal morality moderated this relationship.	*N* = Competitive athletes (Study 1, *N* = 366; Study 2, *N* = 200).	Cross-sectional Quantitative	Online survey, multiple imputation model, Cronbach alpha coefficients, Pearson zero-order correlations	Study 1: Participants competed in a team (54%) and individually (46%) sports at club (40%), university (6%), county (15%), regional (10%), national (22%) and international (6%) level. Study 2: Participants were from team (61%) and individual (39%) sports competing at club (26%), university (8%), country (10%, regional (13%), national (34%) and international (10%) level.	Q1
Hurst et al. (54)	To identify whether dietary supplement use precedes doping and examines what moderates the dietary supplement-doping relationship.	*N* = Competitive athletes (*N*=1,081, 42.0% female. Age=29.3 ± 10.8 years)	Retrospective cross-sectional Quantitative	online survey, Crosstabs, chi-square (χ2), Cramer's V (V) statistics, Paired samples *t*-tests, Pearson zero-order correlations, *post-hoc* power analyses, linear multiple regression model.	Thirty-one different sports were represented within the sample, with track & field (28.5%), football (19.5%) and cycling (9.5%) the most popular.	Q1
Hurst et al. (55)	The aim of the present research is to extend previous work by examining whether the relationship between dietary supplement use and doping is indirectly related with Ajzen's (1985, 1991) theory of planned behaviour.	*N* = Competitive athletes (*N* = 443; 46% female, age = 27.0 ± 8.6 years old, years competing = 8.3 ± 3.5)	cross-sectional Quantitative	Partial Last Squares, Cronbach Alphas, Point Biserial and Pearson zero-order correlations, Bootstrapping, Regressions.	Participants competed in 21 different sports, with the most popular being football (31%), weightlifting (15%) and athletics (12%).	Q2
Kristensen (56)	The main aim of the present study was to examine whether a change in dietary supplement acceptance mediates the effects of supplement use at season start on doping attitudes at the end of the sports season.	*N* = 217 elite youth athletes (47% male; mean age = 16.98 years, standard deviation = 0.88) who competed in team sports and individual sports.	two-wave half-longitudinal. Quantitative	Structural equation modelling (SEM), questionnaires, correlations, chi-square test, comparative fit index (CFI), Tucker–Lewis's index (TLI), root mean square error of approximation (RMSEA), and standardized root mean square residual (SRMR), bootstrapping.	team sports (43%; *N* = 93; basketball, floorball, handball, and ice hockey) and individual sports (57%; *N* = 124; alpine skiing, biathlon, cross-country skiing, swimming, and tennis).	Q1

**Table 3:** Summary of the networks and Key actors studies included in the review.

Autor(s)	Purpose	Participants	Desing	Method(s)	Sport(s)	Quartile
Abreu et al. (57)	Know how nutritionists working with elite soccer teams perceive and use these substances in their daily practice.	*N* = The scope of this work was limited to nutritionists working with soccer players from elite clubs. 65 participants were included.	Cross-sectional Quantitative.	Questionnaire, Shapiro–Wilk Test, The Mann–Whitney test, The Kruskal-Walli's test, Spearman's correlation, The Fisher's exact test, The Freeman-Halton extension.	Elite Soccer Teams from: English Premier League, Spanish La Liga, Italian Serie A, German Bundesliga, French Ligue 1, and Portuguese Primeira Liga) and Brazil (Serie A)	Q1
Boardley et al. (83)	Tested a conceptually grounded model linking athlete perceptions of strength and conditioning and technical coach doping confrontation efficacy (DCE) with athletes’ doping self-regulatory efficacy (SRE), doping moral disengagement (MD), and susceptibility to intentional and inadvertent doping.	*N* = Participants were high-level athletes (male = 532; female = 290) recruited in Australia (*n* = 261), the UK (*n* = 300), and the USA (*n* = 261).	Cross-sectional, correlational. Quantitative.	Questionnaire, Grounded model, Structural equation modelling (SEM), zero-order Pearson's and factor correlations, Bootstrap, chi-square (χ2), comparative fit index (CFI), Tucker-Lewis's index (TLI), and root mean square error of approximation (RMSEA).	Competing at the regional (*n* = 244), national (*n* = 296), or international (*n* = 265) level.	Q1
Boardley et al. (83)	To investigate the nature of doping confrontation efficacy (DCE) beliefs – as well as their antecedents and outcomes – through a qualitative examination of Sullivan, Feltz, LaForge-Mackenzie, and Hwang's (2015) DCE model with high-level technical and strength and conditioning (S&C) coaches from athletics and rugby union.	*N* = Twenty-one coaches participated in the study (male=15, female=6)	Qualitative, descriptive.	Semi-structured interviews, content analysis techniques, coding frame and set of coding rules.	Athletics and Rugby	Q1
Engelberg and Moston (59)	Understand how coaches see their role directly and indirectly influencing the doping attitudes and behaviours of athletes.	Fourteen elite-level coaches participated. (Nine males and five females)	Qualitative	Focus group, topic guide, Analysis Method Framework, Data Index.	American football, Australian Rules Football, basketball, cycling, football, gymnastics, kayaking, netball, rugby union, surf-lifesaving, taekwondo and triathlon	Q1
Fruchart et al. (60)	The present study aimed to map individuals’ ethical positions according to the use of nutritional supplement in sport.	107 adolescents athletes (Mage = 13.64, SD = 1.64) and 157 adults including 44 non-athletes (Mage = 20.56, SD = 2.98), 94 amateur athletes (Mage = 20.61, SD = 2.77), and 19 professional athletes (Mage = 20.52, SD = 2.52	Quantitative	Questionnaire, Cluster analyses, ANOVAs, and chi-square.	N/A	Q1
Ohl et al. (2013)	Understand how the specific interactions between actors involved in the production of performance influence the socialization process by which cyclists learn their job.	*N* = 22 recently professional cyclists, 22 retired cyclists, six coaches, five physicians, 10 team managers, with five other interviews with journalists or policymakers.	Qualitative	Observations, field notes, semi-structured interview, thematic analysis.	Cycling	Q1
Patterson et al. (61)	(1) establish the status of anti-doping education for coaches; (2) gain an understanding of the system through which Anti-doping education is provided to coaches; and (3) explore the opportunities for future education provision. This	*N* = Individuals responsible for anti-doping education within 18 national and international sports or anti-doping organizations were invited to an interview to take place at a time and location chosen by them. In total, 13 stakeholders were interviewed. Male = 9. Female = 4.	Qualitative	Semi-structured interviews, thematic analysis, coding.	national and international sports or anti-doping organizations.	Q1
Skilbred et al. (19)	Explore the actors that contribute to young athletes, meaning making nutritional supplements and anti-doping and examine how the young athletes describe these actors’ contribution.	Twenty-four athletes from private elite upper secondary sport schools (PEUSS) in Norway	Qualitative	Semi-structured interviews, thematic analysis.	Ice hockey, handball, biathlon, motocross, track and field, swimming, cross country, and football.	Q1

**Table 4:** Summary of the grey area of sports supplementation studies included in the review.

Autor(s)	Purpose	Participants	Desing	Method(s)	Sport(s)	Quartile
Haubenstricker et al. (62)	The purpose of the study was to examine how the TPB can explain protein intake and dietary supplement use in-season competitors.	112 in-season competitors.	Cross-sectional Quantitative	Questionnaires, focus group, ANOVA, Sidak multiple comparison testing, Multiple linear regression, Pearson's correlations.	Women bodybuilders: Bikini, Bodybuilder Figure, Fitness, Physique, Wellness.	Q1
Königstein et al. (63)	This study's aim was to analyse the geographical heterogeneity of doping-related knowledge, beliefs and attitude among adolescent elite athletes.	533 athletes (54% females, mean age: 16.0 ± 1.0 years)	Cross-sectional Quantitative	Questionnaires, Cronbach's Alpha, Clusters, ANOVA, *t*-test, multiple regression analyses, Tukey–Anscombe plots, Q–Q plots, variance inflation factors (VIF).	Youth Olympics participants	Q1
Kristensen et al. (32)	This study aimed to systematically review previous studies on contextual and personal factors associated with adolescent athletes’ performance-enhancing and health-compromising behaviours.	N/A	Systematic mixed-studies review	Systematic mixed-studies review, Mixed Methods Appraisal Tool (MMAT).	Young athletes aged 14–20 years.	Q2
Kuff et al. (64)	This study proposes and tests a comprehensive model of gym users’ intention to consume insect-based protein bars and powders, incorporating measures of health, sustainability, taste and perceived risk in the TPB model.	We obtained a total of 256 responses, 55% of which were submitted by men, and 77% were aged between 21 and 40 years; therefore, mostly younger adults.	Quantitative and descriptive study	Questionnaires, structure equation modeling (SEM), average variance extracted (AVE) and maximum shared variance (MSV), Cronbach's alpha, linear equations.	The target population was made up of consumers that regularly attend gyms to practice physical activities and take sports supplements.	Q1
Langan-Evans et al. (65)	The aim of the present study was to gain specific insight into the sport nutritional 55 experiences that females may face during training and competition and examine if this may be influenced by any 56 associated symptomology driven by the menstrual cycle or hormonal contraceptive use.	multiple participants 4 groups (*n* = 18), including athletes (*n* = 7), practitioners (*n* = 6) and researchers (*n* = 5).	Qualitative	Semi-structured interviews, content, thematic analysis, latent themes.	Endurance, team, weight restricted, multisport disciplines.	Q1
Muwonge et al. (66)	The current study set out to establish the doping attitudes, knowledge and practices of professional Ugandan athletes, gathering information that may guide the design of more efficient doping prevention programs.	384 professional Ugandan athletes. The mean age of the athletes was 24 years. The majority of the interviewed athletes were male (60.6%).	Cross-sectional. Quantitative.	Questionnaires, independent sample *t*-test, one way ANOVA, Scheffe's test as *post hoc*.	Team sports (basketball, football, handball and rugby) and two individual sports (athletics and cycling).	Q1
Placentino et al. (67)	The present study investigated the potential motivations to accept an energy protein bar with cricket flour, among a group of selected Italian professional athletes. A second aim was also to measure how an information treatment about the benefits of edible insects would have impact on acceptance	Sixty-one professional athletes (27 females) aged 19 to 39 years.	Quantitative	Questionnaires, Friedman's and Cochran's *Q* test, Cronbach's Alpha coefficient, Pearson's correlation, Spearman’ correlation, one-way-ANOVA, and a Mann–Whitney *U*-test, Levene's test, multiple linear regression analysis, two-way repeated measures ANOVA, *post hoc* dependent *t*-tests, the Wilcoxon signed-rank test and Glass's delta.	Athletes from track and field (31.1%), fencing (23.0%), and beach-volleyball (18%); athletes from archery, sailing, skeet shooting, tennis table and artistic gymnastic.	Q1
Sak et al. (68)	This study aimed to determine cyclists’ nutritional knowledge and habits and nutritional ergogenic aid usage and shed light on the relation between cyclists’ nutritional knowledge and ergogenic aid usage.	174 participated in the presented study. *N* = 58 competitive cyclists-CC, *N* = 58 recreational cyclists-RC, and *N* = 58 control group-CG: sedentary adults).	Quantitative	Questionnaires, Levene test, Pearson Chi-Square, Independent Sample *t*-test, One Way ANOVA, and Post Hoc; Bonferroni test.	Competitive cyclists, recreational cyclists.	Q4
Turfus et al. (69)	The aim of this study was to investigate supplement usage, attitudes towards supplements and knowledge about the World Anti-Doping Code among young Jamaican athletes and to see whether there was a difference in those competing in athletics compared to other sports.	127 Jamaican athletes aged 12–19 years	Quantitative	paper-based surveys, The Fisher's Exact test, Spearman correlation	Champs athletics competition (track and field events), football (*N* = 14), basketball (*N* = 8), boxing (*N* = 1) swimming (*N* = 3), netball (*N* = 13), water polo (*N* = 1), hockey (*N* = 4), tennis (*N* = 1) and dance (*N* = 4).	Q2
Wardenaar et al. (70)	To better understand athlete's awareness and use of third-party supplement testing systems, we further analysed a previously examined data set by assessing the self-reported knowledge and attitudes of Dutch Olympic status and non- Olympic status athletes toward an independent Dutch third-party testing supplement system (NZVT).	*N* = 601 Dutch Olympic status and non-Olympic status Athletes	Quantitative	Questionnaires, Chi square tests (χ2), Mann Whitney *U*-Tests	N/A	Q2
Wirnitzer et al. (71)	The present study aimed to investigate and compare supplement intake between female and male distance runners (10 km, half-marathon, (ultra-marathon) and the potential associations with diet type and race distance.	*N* = 317 Endurance runners. *N* = 127 Females. *N* = 93 Males.	Cross-Sectional Quantitative	Questionaries, Chi square tests (­χ2) nominal scale, Logistic Regression, One-Way ANOVA.	Endurance runners.	Q1
Yasuda et al. (72)	Therefore, this review summarizes how the “meal first” strategy and planned supplement use are important for enhancing athletes’ health and performance.	N/A	Literature Review Consensus Statement.	Literature Review	Japan high performance sport	Q1

**Table 5:** Summary of the conceptual ambiguity studies included in the review.

Autor(s)	Purpose	Participants	Desing	Method(s)	Sport(s)	Quartile
Burke (73)	The aim of This review is to examine our current state of knowledge around these issues. These issues included the additive and interactive effects of combining the use of several performance supplements for a single event, considerations regarding the repeated use of a performance supplement within a relatively brief period, and the notion of individual responsiveness to supplement use.	N/A	Literature Review	literature search	N/A	Q1
Close et al. (74)	The purpose of this narrative review is to explore the concept of the food first approach to sport nutrition with specific targeted supplementation. Finally, guidance will be provided as to how best to deliver a “food first, but not always food only” (abbreviated to FFNFO) sport nutrition strategy in a safe and evidence-based manner.	N/A	Narrative review	literature search	N/A	Q2
Edenfield (75)	For the purposes of this review, we categorize nutritional supplements into 3 categories to include sports foods (gels, bars, drinks, protein powders), medical supplements (vitamins and minerals used to treat clinical deficiency or problem), and ergogenic supplements (used to benefit performance).	N/A	Literature Review	literature search	N/A	Q1
Garthe and Maughan (76)	A discussion around medical, physiological, cultural, and ethical questions may be warranted to ensure that the athlete has the information needed to make an informed choice.	N/A	Evidence-based Consensus Statement.	N/A	N/A	Q2
Maughan et al. (77)	The use of supplements for specific athletic events is covered elsewhere in this issue. This review will focus on some of the general issues relating to the use of dietary supplements and will look in detail at a few supplements that may have something to offer to some athletes.	N/A	Evidence-based Consensus Statement.	literature search	N/A	Q1
Maughan et al. (15)	This review summarizes the issues faced by high-performance athletes and their support team (coach, trainer, nutritionist, physician) when considering the use of supplements, with the goal of providing information to assist them to make informed decisions.	N/A	Evidence-based Consensus Statement.	literature search	N/A	Q2
Molinero and Márquez (78)	Many supplement products contain substances that are prohibited in sport or that have been associated with significant morbidity and mortality. For athletes, lack of knowledge or misinformation has been established despite numerous sources of information being available, and the reasons for, and implications of, unsupervised and unrestricted supplement use require further attention.	N/A	Literature Review	literature search	N/A	Q3
Pascale (4)	Certain dietary supplements (DSs) used by military populations pose a threat to overall readiness. This study assessed members of the American Medical Society for Sports Medicine (AMSSM) regarding their knowledge of DS use among their patients and reporting of suspected adverse events.	Military and civilian sports medicine physicians. 311 physician members of the American Medical Society for Sports Medicine (AMSSM).	Retrospective cross-sectional	Questionnaires, Web based Survey.	N/A	Q2
Skilbred et al. (20)	Our aim with this paper is to explore how athletes make sense of supplements within the specific context of being aspiring youth athletes at a PEUSS in Norway.	total of 24 athletes aged 17–19 years from three private elite upper-secondary sport schools (PEUSS) in Norway participated in this study.	Qualitative	Interviews, semi-structured, frame analysis, meaning making.	Ice hockey, handball, biathlon, motocross, track and field, swimming, cross country and football.	Q1

## 4 Results

This section presents the findings of the systematic review, organised into themes of significance for the social sciences. Each theme is accompanied by the number of articles selected and a brief overview of their definitions and areas of study.

### 4.1 Attitudes towards supplements and doping

This theme, represented by a significant portion of the reviewed literature (*N* = 18), underscores the critical role of social sciences in understanding and informing preventive strategies against doping in sports. It reveals how these disciplines provide essential frameworks for examining the intricate web of motivations, perceptions, and social influences that underlie athletes' supplement use and doping behaviours.

Doping is often conceptualised not merely as a violation of ethical standards, but rather as a complex outcome arising from a multifaceted risk assessment process that athletes engage in, influenced by their environments. This perspective shifts the focus from individual moral failings to systemic factors that shape athlete behaviour.

The studies within this theme explore an extensive array of topics, including, but not limited to, the following: how social and cultural factors shape athletes' attitudes towards supplements; the knowledge athletes possess regarding the risks and benefits associated with their use; and the moral frameworks that guide their decision-making processes. The diverse cultural contexts in which athletes operate play a significant role in shaping their beliefs about supplements, often leading to varied interpretations of what constitutes acceptable or unacceptable practices within the sporting community.

Moreover, this research encompasses a wide range of demographics, spanning populations from elite professional athletes to younger amateur enthusiasts, including adolescents. By examining these different groups, the literature offers nuanced insights into the social fabric that influences decisions regarding supplement use and, at times, the transition towards doping.

The identification of common themes emerging from these studies illustrates how athletes navigate their environments, weighing perceived benefits against potential risks. As pressures from coaches, peers, and commercial interests mount, athletes often grapple with conflicting messages that complicate their understanding of ethical choices and health implications. Furthermore, this body of work emphasises the need for tailored interventions that account for the unique social, cultural, and psychological factors influencing each subgroup, highlighting the importance of contextualised education and support systems.

Ultimately, the exploration of attitudes towards supplements and doping through the social sciences not only enhances our understanding of athlete behaviour but also serves as a foundation for developing effective prevention strategies. These insights are invaluable for stakeholders—ranging from sports governing bodies to educational institutions—seeking to foster a culture of clean sport and ethical competition.

### 4.2 Gateway to doping

The gateway hypothesis suggests that the consumption of nutritional supplements may serve as a precursor to the use of prohibited doping substances. This notion reflects a pattern observed in recreational drug use, where the intake of “soft” substances often leads individuals towards the use of “harder” drugs. In the realm of sports supplementation, the literature within social sciences has robustly developed this hypothesis through various complementary explanatory mechanisms.

One of the key frameworks is the Incremental Doping Behaviour Model, which underscores the motivated progression towards performance-enhancing methods (53). Within this model, athletes may begin their journey with legal supplements, creating a context in which the allure of performance enhancement becomes increasingly appealing.

Additionally, the literature delineates several other mechanisms that facilitate transitions between supplement use and doping. One significant aspect includes processes of justification and rationalisation that enable athletes to reconcile their use of various substances (48). These processes can create an environment where athletes feel justified in their choices, despite the ethical implications.

Subjective norms also play a crucial role as moderators in this shift towards doping, influencing athletes' perceptions of what is acceptable behaviour within their sporting context (54, 55). The surrounding culture, including peer and coach expectations, can significantly impact an athlete's decisions, often pushing them towards doping as a seemingly normative behaviour.

Another critical concept within this discourse is the idea of “licensing unhealthy lifestyles.” This phenomenon can lead athletes to engage in riskier behaviours, as they may perceive that their use of supplements entitles them to take further risks, including the potential use of prohibited substances (48).

Moreover, specific predictive variables for the consumption of prohibited substances have been identified in various studies. These include factors such as athletes' beliefs about the effectiveness of such substances, their perceptions of safety, and the socio-cultural environment influencing their choices (50, 56).

This theme is represented by 9 of the 56 articles included in the systematic review, illustrating its significance within the broader context of sports supplementation and doping behaviours. Understanding the dynamics encapsulated by the gateway hypothesis is essential for identifying intervention points that can effectively deter athletes from harmful supplementation practices that might lead to doping.

### 4.3 Networks and key actors

This theme emphasises the collective understanding of the systems that influence supplement intake and practices associated with sports performance. The studies examine the intricate interplay among individuals and institutions that comprise the social environment surrounding athletes. Researchers illuminate specific configurations that form what some authors refer to as “socialisation ecosystems,” “social drama of work,” or the concept of the “networked athlete.”

Within these networks, key actors—such as technical and strength coaches, medical personnel, nutritionists, teammates, family members, and representatives from anti-doping organisations—exert considerable influence over athletes' decisions. These actors play pivotal roles in shaping athletes' attitudes towards supplementation and performance enhancement, significantly impacting their choices and behaviours in the pursuit of improved performance.

For instance, coaches are not only responsible for training but also for establishing norms around supplement use within their teams. Their beliefs and practices can create an environment where specific supplements are either encouraged or discouraged. Similarly, medical personnel and nutritionists provide guidance on the safe and effective use of supplements, and their recommendations can significantly shape athletes' perceptions of the necessity and safety of these products.

Teammates and peers often reinforce or challenge this guidance, further complicating the decision-making process. The influence of family members, who may provide emotional support or financial backing, also plays a critical role in shaping athletes' approaches to supplementation. Finally, anti-doping organisations are crucial in communicating the risks associated with prohibited substances and promoting a culture of clean sport.

This theme is supported by 8 articles selected from the 56 texts reviewed, highlighting its significance within the broader discourse on sports supplementation. Understanding these social networks and key actors is essential for developing targeted interventions to promote healthy supplementation practices and prevent doping behaviours among athletes. By recognising the multifaceted influences on athletes’ decisions, stakeholders can foster environments that prioritise ethical practices and informed decision-making.

### 4.4 Grey area of sports supplementation

This theme delves into the uncertainties surrounding scientific knowledge regarding sports supplements and their usage. As the landscape of sports nutrition evolves, research in this field seeks to bridge existing knowledge gaps and clarify conceptual vagueness through empirical studies focused on specific populations that have historically been underrepresented or vulnerable.

A fundamental area of investigation involves examining previously understudied populations, particularly adolescent athletes. Königstein et al. (63) highlighted significant gaps in knowledge about doping-related attitudes and beliefs among this demographic. They found that many young athletes are unaware of the risks associated with certain supplements, underscoring the need for tailored educational interventions to enhance understanding within this group.

Furthermore, studies indicate knowledge disparities among different athlete groups. Haubenstricker et al. (62) explored the influence of social dynamics on supplement use among competitive female bodybuilders. Their findings reveal that cultural and social contexts heavily shape athletes' beliefs and practices regarding supplementation, illustrating the vital role of subjective experiences in making informed decisions.

Moreover, the tension between current supplementation practices and the available scientific evidence is significant. Research by Kristensen et al. (56) identified that many athletes operate with misconceptions about the efficacy and safety of various supplements, leading to reliance on anecdotal information rather than evidence-based guidelines. This disconnect highlights the critical need for trustworthy information and education aimed at mitigating these gaps.

The complexities surrounding regulation and the integrity of supplement products further complicate matters. Studies like those by Muwonge et al. (66) note that athletes, particularly adolescents, are at risk of consuming potentially contaminated or mislabelled products, thereby jeopardising their health and eligibility. This issue is particularly pressing given the lack of robust regulatory frameworks governing the supplement industry.

By focusing on these 12 articles, it becomes evident that understanding the uncertainties of sports supplement use is essential for developing effective interventions and educational strategies. Researchers, policymakers, and sports professionals must address these grey areas to promote safer, more informed practices among athletes—ultimately fostering a culture of integrity and sound decision-making regarding supplementation.

### 4.5 Conceptual ambiguity

This section highlights the prominent lack of a standard definition and classification of sports supplements, a challenge with significant implications across various levels of sports science and practice. Research underscores that the lack of consensus on what constitutes a sports supplement complicates not only scientific methodology but also the broader applicability of research findings and policy recommendations across diverse cultural and regulatory landscapes.

Burke (73) discusses how differing regulatory frameworks in various countries contribute to this conceptual ambiguity. Variation in definitions can lead to regulatory disparities that hinder effective communication about what is acceptable in different contexts. For instance, countries may classify certain supplements as food while others might categorise them as drugs, creating confusion for athletes who may not understand the legal implications of their supplement choices.

Additionally, Close (74) points to the challenges of integrating scientific evidence with real-world applications. Practical complications arise, particularly for athletes and professionals engaged in decision-making around supplementation. What one athlete considers a legal supplement, another might view as bordering on doping. This variability underscores the crucial need for a clear, universally accepted framework that distinguishes between beneficial supplements and prohibited substances.

Furthermore, Königstein et al. (63) examine how cultural perceptions of supplements can exacerbate these ambiguities. In certain sports cultures, the use of specific supplements may be normalised, despite their unclear safety and legal status. This pervasive ambiguity also complicates classification, contributing to conceptual vagueness in the industry, where distinctions between food, drugs, and supplements are increasingly blurred.

Moreover, many athletes acquire supplements through informal channels—such as gyms, stores, and online platforms—often without adequate guidance or interaction with healthcare professionals (62). This lack of professional oversight can lead to unintentional consumption of harmful or banned substances, complicating their health and eligibility.

This theme comprises 9 articles selected from the 56 texts included in the systematic review, underscoring the urgency to address the underlying issues of definitional and regulatory heterogeneity. A clear understanding of what constitutes a supplement is necessary not only for the integrity of research but also for ensuring athletes can make informed decisions about their health and performance.

## 5 Discussion

The subsequent discussion will interpret the findings from our five thematic categories by drawing upon established social science frameworks. This approach directly addresses the “intersectional phenomenon” of sports supplementation, leveraging insights from social psychology, sociology of sport, science and technology studies, and ethical theory. By integrating these theoretical perspectives, we aim to provide a more rigorous and nuanced understanding of the social, cultural, and ethical dimensions influencing athletes' decisions and perceptions regarding supplementation.

This review unveils a complex landscape of attitudes, behaviours, and perceptions among athletes regarding supplements and doping, highlighting a critical area of ongoing concern within social science. A valuable reference is the International Olympic Committee (IOC) recommendation, which defines a supplement as a food, food component, nutrient, or non-food compound deliberately consumed in addition to a regular diet to attain specific health or performance advantages. Supplements are categorised based on their form, including functional foods enriched with nutrients, formulated and sport-specific foods that provide convenient sources of energy and nutrients, isolated nutrients and herbal compounds, and multi-ingredient products that combine these elements. In sports nutrition, their primary roles involve addressing micronutrient deficiencies, providing convenient sources of energy and macronutrients, and offering direct or indirect performance benefits such as enhanced recovery, physical conditioning, or mental well-being.

Scientifically, the evidence supporting the effectiveness of these supplements is ranked within a hierarchy, with systematic reviews and meta-analyses at the top due to their comprehensive synthesis of multiple studies. These reviews provide broad, conclusive insights into efficacy but are limited by the quality of the underlying studies. The most rigorous method to determine a supplement's true impact on performance is through well-designed, prospective, randomised controlled trials (RCTs), ideally double-blinded and standardised. This hierarchical structure aims to distinguish between anecdotal or mechanistic hypotheses and scientifically validated effects, guiding evidence-based recommendations.

The IOC's definition emphasises a dual taxonomical approach to categorising sports supplements, considering both their form and purpose. It underscores that the most substantial scientific evidence comes from high-quality RCTs, with systematic reviews and meta-analyses providing an overarching insight. Nonetheless, most current knowledge relies on lower-tier evidence such as athlete observations and scientific hypotheses. To truly understand the effects of supplements on athletic performance, meticulous scientific research following the gold standard of RCTs under standardised conditions is essential, ensuring that conclusions are both valid and applicable to real-world sports settings.

Central to this discussion is the recognition that the prevalent use of dietary supplements is strongly associated with the desire to optimise performance, control weight, and gain a competitive advantage (41). However, these attitudes are deeply embedded within complex belief systems shaped by cultural, social, and individual factors, with knowledge levels about supplement efficacy and safety varying markedly among athletes. This variation underscores the importance of accessible, accurate information in preventing decisions influenced by misconceptions or incomplete understanding (46). Attitudes and perceptions are further heavily influenced by media, coaches, peers, and the wider sporting environment, which can both support and undermine ethical practices and fair competition (18).

A significant concern that intersects with these attitudes is the persistent issue of doping, especially among adolescent athletes (42, 45). Various personal, situational, and contextual factors contribute to the initiation of doping behaviours, emphasising the importance of early intervention programmes tailored to at-risk groups (34). The concept of moral disengagement plays a pivotal role here, as it allows athletes to justify the use of prohibited substances by minimising the moral seriousness of their actions or blaming external factors (36, 58). Such rationalisations often stem from distorted beliefs about success in sport—that doping is viewed erroneously as the only pathway to achievement—leading young athletes to adopt an anti-ethics stance based on social and cultural norms. Attitudes such as “everyone is doing it” or perceptions that doping is a necessary evil appear to be reinforced by social pressures, including team expectations and coach influence, potentially shaping behaviour in ways that can be difficult to alter (44).

Furthermore, the influence of societal norms and the cultural environment significantly affect athlete behaviour. Cross-cultural studies indicate that moral perceptions and attitudes towards doping vary considerably depending on geographic and cultural contexts, which necessitate tailored prevention strategies (56, 63). Prevention efforts should integrate education on ethics and integrity, especially in young athletes, fostering resilience through programmes that promote ethical decision-making and autonomous motivation (43). Additionally, fostering self-assertiveness and self-efficacy has emerged as a promising approach to counteract social pressures and reduce susceptibility to doping (34). These strategies are vital for cultivating a sporting culture rooted in fairness and respect for clean competition, addressing the underlying social and personal determinants that influence doping behaviour.

Within this context, a noteworthy aspect is the so-called “grey area” of sports supplementation, a domain marked by ambiguity in regulation, knowledge, and usage. Despite the substantial participation of women in both recreational and elite sports, research on gender-specific aspects remains scarce, resulting in a gender gap in understanding and guidelines (65, 71). Studies comparing male and female athletes reveal that women report slightly higher supplement usage, often driven by specific needs such as menstrual symptom management, which traditional guidelines do not adequately address (71). A deeper understanding of gender differences in supplement practices is critical, especially since current recommendations are primarily based on data from male athletes, creating a knowledge imbalance.

Adding to this complexity is the pervasive lack of knowledge among athletes and coaches regarding the risks and benefits of supplement use, a situation compounded by inconsistent regulation and widespread availability of unverified products (66, 69). The grey area is further highlighted by inconsistencies in definitions, regulatory oversight, and labelling, which often result in contaminated or mislabelled products that may contain banned substances or pose health risks. The real-world application of supplements, their perceived safety, and their potential to act as gateways to doping further complicate this picture (Garthe & Maughan; Paulsen et al.). The concept of the “borderline” status of some supplements means athletes frequently navigate a “zone of uncertainty”, trying to balance the benefits perceived from supplements against the risks of contamination, doping violations, or adverse health effects.

Crucially, the hypothesis that supplement use is associated with doping behaviour has gained empirical support, with evidence showing that supplement consumption correlates with favourable attitudes towards doping, and in some cases, precedes the initiation of doping practices (49, 51, 56). Studies have demonstrated that beliefs about the safety, necessity, and normalisation of supplement use are often linked to athletes' likelihood of engaging in doping—both intentionally and inadvertently—particularly when they perceive supplements as part of routine performance enhancement (50, 52).The realising concern is that some athletes view supplement use as a normalised aspect of training, a perception that may be associated with weakened moral boundaries and an increased likelihood of accepting or even initiating doping behaviours. Additionally, the complex interplay between the social environment, regulatory gaps, and individual beliefs often creates a “culture of risk,” where the ambiguity surrounding supplement and doping policies exacerbates the challenge of preventing illicit practices in sport.

The role of key stakeholders, such as coaches, support staff, and institutional actors, further compounds this complex web. Historically, responsibility for doping prevention has been placed solely on the athlete, overlooking the broader social network that influences athlete behaviour (59). Recent research emphasises that coaches and support personnel, including physiotherapists, dietitians, and strength and conditioning professionals, hold significant influence over athletes' decisions and perceptions regarding doping and supplement use (58). Notably, the level of trust and confidence between coaches and athletes is crucial; deficiencies in confidence and communication can diminish the effectiveness of education programmes and intervention strategies. Consequently, developing trustful relationships, fostering open dialogue, and promoting ethical standards within the support network become fundamental in curbing doping practices.

Furthermore, the social production of norms and the embedded culture within sporting environments play a crucial role in shaping the behaviour of athletes. Research using sociological and discursive approaches reveals how athletes often perform social identities within their teams and organisations, balancing notions of fairness, competitiveness, and moral conduct (19, 84). The social environment, coupled with organisational norms and institutional policies, can either reinforce anti-doping messages or contribute to a permissive culture where illicit practices are considered acceptable or inevitable.

The integrity of the supplement industry itself remains a concern. The “grey area” extends beyond social influences to regulatory shortcomings, with many products available on the market contaminated with banned substances or mislabelled, thus blurring the boundaries between legal supplement use and doping (70, 72). The lack of third-party verification, inconsistent international regulation, and widespread availability of untested products create a milieu where athletes are at constant risk of inadvertent doping violations. Efforts, such as those by third-party testing organisations and national regulatory programmes, aim to mitigate these issues, but gaps still persist. Given the potential gateway effect of supplements, where their use is associated with increased familiarity with performance enhancement and can foster attitudes favourable to doping, it remains critical that policies, education, and industry standards evolve towards greater transparency, regulation, and athlete support.

Finally, attitudes and behaviours towards supplements and doping are deeply intertwined, influenced by social, cultural, psychological, and institutional factors. The grey areas—marked by regulatory ambiguity and knowledge gaps—constitute significant barriers to effective prevention. Addressing these challenges requires a comprehensive approach that involves education, fostering ethical norms, strengthening stakeholder networks, and enhancing regulatory oversight. As research continues to shed light on these complex dynamics, targeted interventions must aim not only to inform but to fundamentally change the culture surrounding performance enhancement in sport, promoting clean competition rooted in fairness, integrity, and athlete well-being.

## 6 Limitations

We recognise that our systematic review, while comprehensive within its defined parameters, has certain limitations. Primarily, the decision to include only studies published in English or Spanish, though a practical necessity for ensuring accurate and nuanced interpretation by the review team, inevitably introduces linguistic and, potentially, cultural bias. This choice means that valuable social science research on sports supplementation conducted and published in other languages and cultural contexts may not have been captured. While our search leveraged major international databases (Web of Science, Scopus, PubMed), which tend to have a broad reach and often include English abstracts for non-English publications, a significant body of local or regional research published in other languages might have been missed. Consequently, the themes and insights presented, while robust for the included literature, may not fully represent the global diversity of social, cultural, and ethical considerations surrounding sports supplementation. Future research endeavours, particularly those aiming for a broader global perspective, would benefit from incorporating a wider array of languages and engaging multilingual research teams to enrich the cultural breadth and generalizability of the findings.

Furthermore, our review's explicit focus on athletic populations (encompassing both recreational and elite levels, as per our inclusion criteria) means that the findings, particularly regarding attitudes, motivations, and the “gateway to doping” phenomenon, may have limited generalizability to the broader public. While the abstract mentions “the general population” in the context of supplement use growth, the subsequent detailed analysis in the results and discussion is predominantly framed around the athlete experience. Many non-athletes also consume dietary supplements for various reasons (e.g., health, wellness, appearance), and their decision-making processes, social influences, and perceptions of risk/benefit might differ significantly from those of individuals engaged in competitive sports. Therefore, the applicability of our conclusions to the broader public who use supplements should be considered with caution. Future research could broaden this scope to explicitly explore the social science dimensions of supplementation across diverse user groups, including the general population, to provide a more holistic understanding of this complex phenomenon in contemporary society.

## 7 Conclusions

This systematic review has explored the complex and often contested realm of sports supplementation through a critical social science lens, unveiling its intricate social, cultural, and ethical dimensions. A key challenge identified is the persistent absence of a universally accepted definition and taxonomy of sports supplements, which hinders rigorous scientific research, confuses athletes and professionals, and complicates regulatory efforts. To address this, our review culminates in a conceptualisation of a supplement within this social science context:

A supplement is best understood as a cultural and commercial product embedded within consumption practices aimed at optimising the body, health, and/or performance. It functions as a mediator between normative ideals—such as productivity, youth, beauty, or self-control—and subjective experiences of well-being and identity. It operates within practices in which individuals internalise responsibility for body management, in contexts where scientific evidence, marketing, and digital communities collaboratively produce “practical truths” about its effects. As a symbolic commodity, the supplement articulates aspirations for personal improvement with neoliberal logics of self-entrepreneurship, generating distinctions of class, gender, and cultural capital through its access, brands, and modes of use, and reconfiguring relationships between expert knowledge, consumer autonomy, and state regulation.

This conceptual clarity serves as a crucial starting point for addressing the multifaceted issues identified across our five thematic areas: attitudes towards supplements and doping, the gateway to doping, networks and key actors, the grey area of supplementation, and conceptual ambiguity itself. The inherent heterogeneity in study designs (qualitative, quantitative, experimental) and athlete populations (elite vs. recreational, and with diverse backgrounds) within social science research, far from being a limitation, precisely illuminates the diverse social contexts and influences that necessitate tailored, rather than monolithic, interventions. This diversity enriches our interpretative depth and the ecological validity of our findings, offering a holistic understanding of this complex phenomenon.

The review further underscores the multifaceted web of influences that shape athletes' attitudes and behaviours towards both sports' supplementation and doping. These influences extend far beyond simple biological or physiological considerations, encompassing a complex interplay of psychosocial factors (such as self-esteem, body image concerns, and pressure to perform), deeply ingrained cultural norms within specific sporting subcultures (related to competitiveness, risk-taking, and acceptable performance enhancement strategies), and the significant role of social networks and key actors (including coaches, peers, family members, and sports nutrition companies) in shaping these dynamics. The review emphasises the importance of understanding these social ecosystems to address problematic supplementation practices.

To effectively navigate this intricate landscape, we propose the following actionable insights for various stakeholders.

*For athletes:*

Cultivate critical literacy: athletes must be empowered to critically evaluate information sources, particularly social media and commercial narratives, regarding supplement efficacy and safety, rather than passively accepting “practical truths”.

Seek integrated professional guidance: prioritise consultation with a multi-disciplinary support network (coaches, medical staff, nutritionists) who can offer evidence-based advice tailored to individual needs, moving beyond a sole reliance on self-driven decisions.

*For coaches and support teams:*

Enhance ethical education: integrate comprehensive training on ethical decision-making, moral disengagement, and the fine line between supplementation and doping into coaching certifications and athlete development programmes.

Foster trust and open communication: build environments of trust where athletes feel safe discussing supplement use and concerns without fear of judgment, thereby strengthening anti-doping messages and promoting clean sport cultures.

*For regulators and policymakers:*

Develop context-sensitive regulations: address the identified “grey area” of supplementation by working towards more precise, more harmonised definitions and regulatory frameworks that account for cultural, social, and commercial dimensions beyond purely biomedical standards. Our proposed conceptualisation can serve as a valuable reference point for this effort.

However, translating these insights and recommendations into effective, real-world implementation is not without significant challenges. Resource limitations, often prevalent in sports organisations and educational bodies, can constrain the development and delivery of comprehensive educational programs, robust verification mechanisms, and consistent regulatory enforcement. Institutional resistance to changing ingrained practices, deeply rooted cultural norms within sporting environments, and the complexities of coordinating diverse stakeholders (including athletes, coaches, medical staff, sports organisations, and commercial entities) often impede the systematic adoption of new, evidence-based strategies. Furthermore, the pervasive influence of social media and commercial narratives, which frequently promote unverified products and misleading claims, poses a continuous challenge to fostering critical literacy and dispelling misinformation among athletes and the wider public. Successfully addressing these deeply entrenched barriers requires not only clear recommendations but also a sustained, collaborative effort to overcome systemic inertia and secure the necessary political will and financial resources for sustained change.

Strengthen oversight of marketing and product integrity: implement robust mechanisms for third-party verification of supplements and stricter controls on misleading marketing claims, especially those targeting vulnerable populations, to mitigate the risks of contamination and misinformation.

*For researchers in social sciences:*

Advance contextualised methodologies: future research should continue to explore the diverse experiences of athletes, particularly those from underrepresented populations (e.g., women, athletes with disabilities, athletes from diverse cultural backgrounds, and various socio-economic groups), using varied qualitative and mixed-methods approaches and further developing tools appropriate to the complexities of the social sciences.

Theorise beyond description: while this review characterises conceptual ambiguity, future social science research is encouraged to develop and test theoretical models that can offer alternative definitions and frameworks for understanding supplementation, thereby fostering greater conceptual clarity and facilitating interdisciplinary dialogue.

Ultimately, this review underscores that effectively addressing the multifaceted complexities of sports supplementation requires a holistic, integrated approach. By fostering a culture of informed decision-making, promoting critical thinking, and emphasising the importance of fair play, the sports community can better navigate the challenges posed by both legitimate supplementation practices and the ever-present threat of doping.

## Data Availability

The original contributions presented in the study are included in the article/Supplementary Material, further inquiries can be directed to the corresponding author.

## References

[1] Peeling P Binnie MJ Goods PSR Sim M Burke LM. Evidence-Based supplements for the enhancement of athletic performance. Int J Sport Nutr Exerc Metab. (2018) 28(2):178–87. 10.1123/ijsnem.2017-0343 29465269

[2] Antonio J Pereira F Curtis J Rojas J Evans C. The top 5 can’t-miss sport supplements. Nutrients. (2024) 16(19):3247. 10.3390/nu16193247 39408214 PMC11479151

[3] Arnaoutis G. Navigating nutritional challenges on the way to Maximum athletic performance. Nutrients. (2024) 16(24):4385. 10.3390/nu16244385 39771006 PMC11678316

[4] Pascale B Steele C Attipoe S O’Connor FG Deuster PA. Dietary supplements: knowledge and adverse event reporting among American medical society for sports medicine physicians. Clin J Sport Med. (2016) 26(2):139–44. 10.1097/jsm.0000000000000213 26035683

[5] Muñoz-Maldonado GE Ochoa-Ahmed FA Díaz-Ochoa EA Ramírez-Orozco RE Gómez Renaud VM. Suplementos deportivos: ¿cómo definimos a estos productos? Lux Médica. (2021) 16(48):1–11. 10.33064/48lm20213235

[6] Zolkepli IA Omar A Ab Rahim NH Tahir SNKM Tiwari V. Social media advertising, celebrity endorsement, and electronic word-of-mouth effect on health supplement purchasing behaviour. Asian J Res Bus Manag. (2023) 4(4):185–99. 10.55057/ajrbm.2022.4.4.15

[7] Kumar N Nawaz Z Samerguy P. The power of social media fitness influencers on supplements: how they affect buyer’s purchase decision? Int J Pharmaceut Healthcare Market. (2024) 18(1):27–46. 10.1108/IJPHM-04-2022-0037

[8] Neswald E Smith DF Thoms U. Introduction. In: Neswald E Smith DF Thoms U Neswald E Thomas U, editors. Setting Nutritional Standards: Theory, Policies, Practices. introduction. New York: Boydell & Brewer (2017). p. 1–28.

[9] Medrano Echeverría M Cadenas-Sánchez C Alfaro-Magallanes VM Labayen Goñi I. Nutrición deportiva (2024).

[10] Boyce EG. Use and effectiveness of performance-enhancing substances. J Pharm Pract. (2003) 16(1):22–36. 10.1177/0897190002239630

[11] Reardon C Creado S. Drug abuse in athletes. Subst Abuse Rehabil. (2014) 95:95–105. 10.2147/sar.s53784 PMC4140700 25187752

[12] Carollo A Corazza O Mantovani M Silvestrini N Rabin O Esposito G. Performance-enhancing substances in sport: a scientometric review of 75 years of research. Drug Test Anal. (2025) 17(1):13–24. 10.1002/dta.3677 38491903 PMC11730354

[13] World Anti-Doping Agency. World Anti-Doping Code international standard: Prohibited list 2025 (2025). Available online at: https://www.wada-ama.org/en/prohibited-list (Accessed June 15, 2025).

[14] Martínez-Sanz J Sospedra I Ortiz C Baladía E Gil-Izquierdo A Ortiz-Moncada R. Intended or unintended doping? A review of the presence of doping substances in dietary supplements used in sports. Nutrients. (2017) 9(10):1093. 10.3390/nu9101093 28976928 PMC5691710

[15] Maughan RJ Burke LM Dvorak J Larson-Meyer DE Peeling P Phillips SM IOC Consensus statement: dietary supplements and the high-performance athlete. Br J Sports Med. (2018) 52(7):439–55. 10.1136/bjsports-2018-099027 29540367 PMC5867441

[16] Singh M Givens M Singh T Rao P. Performance enhancing supplements and cardiovascular health. Curr Treat Options Cardio Med. (2025) 27:29. 10.1007/s11936-025-01086-2

[17] Ohl F Fincoeur B Lentillon-Kaestner V Defrance J Brissonneau C. The socialization of young cyclists and the culture of doping. Int Rev Sociol Sport. (2015) 50(7):865–82. 10.1177/1012690213495534

[18] Szűcs RS Szakály Z. The perception of dietary supplements among consumers engaged in sports on a regular basis. Corvinus J Soc Soc Pol. (2020) 11(2):99–118. 10.14267/cjssp.2020.2.6

[19] Skilbred A Loland S Strandbu A. Young networked athletes and performance-enhancing substances: who are the actors in their network, and how do the actors shape athletes’ meaning-making? Eur J Sport Soc. (2024) 21(4):317–36. 10.1080/16138171.2024.2335578

[20] Skilbred A Strandbu Å Loland S. Youth athletes’ framing of nutritional supplements: performance enhancement and food. Int Rev Sociol Sport. (2025) 60(1):45–63. 10.1177/10126902241260923

[21] Backhouse SH Whitaker L Patterson L Erickson K McKenna J. Social psychology of doping in sport: a mixed-studies narrative synthesis. (2016). p. 1–263. Available online at: https://www.wada-ama.org/sites/default/files/resources/files/literature_review_update_-_final_2016.pdf

[22] Manterola C Rivadeneira J Delgado H Sotelo C Otzen T. ¿Cuántos Tipos de Revisiones de la Literatura Existen? Enumeración, Descripción y Clasificación. Revisión Cualitativa. Int J Morphol. (2023) 41(4):1240–53. 10.4067/s0717-95022023000401240

[23] Hong QN Fàbregues S Bartlett G Boardman F Cargo M Dagenais P The mixed methods appraisal tool (MMAT) version 2018 for information professionals and researchers. Educ Inform. (2018) 34(4):285–91. 10.3233/EFI-180221 (Original work published 2018).

[24] Rouleau G Hong QN Kaur N Gagnon M Côté J Bouix-Picasso J Systematic reviews of systematic quantitative, qualitative, and mixed studies reviews in healthcare research: how to assess the methodological quality of included reviews? J Mix Methods Res. (2021) 17(1):51–69. 10.1177/15586898211054243 (Original work published 2023).

[25] Porta M. A Dictionary of Epidemiology. New York: Oxford University Press (2014). Available online at: https://www.oxfordreference.com/view/10.1093/acref/9780199976720.001.0001/acref-9780199976720 (Accessed June 15, 2025).

[26] Tavory I. Deceptively approachable: translating standards in qualitative research. Sociol Methods Res. (2023) 52(2):1043–7. 10.1177/00491241221140431

[27] Small ML Calarco JM. Qualitative Literacy: A Guide to Evaluating Ethnographic and Interview Research. Oakland, CA: Univ of California Press (2022).

[28] Navarrete C Campos F Ojeda Pereira I. Opening the methodological black box in science and technology studies of the future(s). shadows and proposals. Futures. (2025) 175:103713. 10.1016/j.futures.2025.103713

[29] McGranahan C. Ethnography beyond method: the importance of an ethnographic sensibility. Sites: a J So Anthropol Cultur Stud. (2018) 15: 10.11157/sites-id373. 10.11157/sites-id373

[30] Quirós J. Etnografiar Mundos Vívidos. Desafíos de trabajo de campo, escritura y enseñanza en antropología; Colegio de Graduados en Antropología de la República Argentina; Publicar en Antropología y Ciencias Sociales; 17; 12-2014; 47-65.

[31] Navarrete C. Rearticulating realism: chains of reference and epistemic success in the sciences. Sociological Theory. (2024) 42(4):354–79. 10.1177/07352751241291831 (Original work published 2024).

[32] Kristensen JÅ Skilbred A Abrahamsen FE Ommundsen Y Loland S. Performance-enhancing and health-compromising behaviors in youth sports: a systematic mixed-studies review. Perform Enhanc Health. (2022) 10(4):100237. 10.1016/j.peh.2022.100237

[33] Barkoukis V Lazuras L Tsorbatzoudis H. Beliefs about the causes of success in sports and susceptibility for doping use in adolescent athletes. J Sports Sci. (2014) 32(3):212–9. 10.1080/02640414.2013.819521 24016156

[34] Barkoukis V Kartali K Lazuras L Tsorbatzoudis H. Evaluation of an anti-doping intervention for adolescents: findings from a school-based study. Sport Manag Rev. (2016) 19(1):23–34. 10.1016/j.smr.2015.12.003

[35] Barkoukis V Harris PR Rowe R Lazuras L. Self-Affirmation and image/performance enhancing drug use in recreational exercise. Res Q Exerc Sport. (2023) 94(3):698–706. 10.1080/02701367.2022.2046253 35452365

[36] Boardley ID Grix J Harkin J. Doping in team and individual sports: a qualitative investigation of moral disengagement and associated processes. Qualitative research in sport. Exerc Health. (2015) 7(5):698–717. 10.1080/2159676x.2014.992039

[37] Boardley ID Smith AL Mills J Grix J Wynne C Wilkins L. Development of moral disengagement and self-regulatory efficacy assessments relevant to doping in sport and exercise. Psychol Sport Exerc. (2018) 36:57–70. 10.1016/j.psychsport.2018.01.007

[38] Campian MD Flis AE Teramoto M Cushman DM. Self-Reported use and attitudes toward performance-enhancing drugs in ultramarathon running. Wilderness Environ Med. (2018) 29(3):330–7. 10.1016/j.wem.2018.04.004 30227921

[39] Chan DKC Hardcastle SJ Lentillon-Kaestner V Donovan RJ Dimmock JA Hagger MS. Athletes’ beliefs about and attitudes towards taking banned performance-enhancing substances: a qualitative study. Sport. Exerc Perform Psychol. (2014) 3(4):241–57. 10.1037/spy0000019

[40] Chan DKC Hardcastle S Dimmock JA Lentillon-Kaestner V Donovan RJ Burgin M Modal salient belief and social cognitive variables of anti-doping behaviours in sport: examining an extended model of the theory of planned behaviour. Psychol Sport Exerc. (2015) 16:164–74. 10.1016/j.psychsport.2014.03.002

[41] Christensen S Gjelstad A Björnsdottir I Lauritzen F. Motivations for using dietary supplements in elite ice hockey—controlling weight and enhancing performance. Nutrients. (2024) 16(16):2667. 10.3390/nu16162667 39203804 PMC11357155

[42] Duncan LR Hallward L Alexander D. Portraits of adolescent athletes facing personal and situational risk factors for doping initiation. Psychol Sport Exerc. (2018) 39:163–70. 10.1016/j.psychsport.2018.08.012

[43] Elbe A-M Brand R. The effect of an ethical decision-making training on young Athletes’ attitudes toward doping. Ethics Behav. (2016) 26(1):32–44. 10.1080/10508422.2014.976864

[44] Kim T Kim YH. Korean National athletes’ knowledge, practices, and attitudes of doping: a cross-sectional study. Subst Abuse Treat Prev Policy. (2017) 12:1. 10.1186/s13011-017-0092-7 28196542 PMC5309985

[45] Mudrak J Slepicka P Slepickova I. Sport motivation and doping in adolescent athletes. PLoS One. (2018) 13(10):e0205222. 10.1371/journal.pone.0205222 30286200 PMC6171920

[46] Sadek Z Mohsen H Yazbek S Nabulsi ZAA Rifai Sarraj A Hoteit M. Dietary supplements use among athletes in Lebanon: knowledge, attitudes, practices, and correlates. Foods. (2022) 11(10):1521. 10.3390/foods11101521 35627091 PMC9140456

[47] Whitaker L Petróczi A Backhouse SH Long J Nepusz T. The role of the self in assessing doping cognition: implicit and explicit measures of athletes’ doping-related prototype perceptions. Psychol Sport Exerc. (2016) 24:159–67. 10.1016/j.psychsport.2016.02.005

[48] Barkoukis V Lazuras L Lucidi F Tsorbatzoudis H. Nutritional supplement and doping use in sport: possible underlying social cognitive processes. Scand J Med Sci Sports. (2015) 25:6. 10.1111/sms.12377 25556707

[49] Barkoukis V Rowe R Harris PR Lazuras L. Self-affirmation effects on doping-related cognition among exercisers who use nutritional supplements. Psychol Sport Exerc. (2020) 46:101609. 10.1016/j.psychsport.2019.101609

[50] García-Grimau E De La Vega R De Arce R Casado A. An explanatory model of doping susceptibility examining morality in elite track and field athletes: a logistic regression analysis. Sustainability. (2022) 14(24):16404. 10.3390/su142416404

[51] Hurst P Kavussanu M Boardley I Ring C. Sport supplement use predicts doping attitudes and likelihood via sport supplement beliefs. J Sports Sci. (2019) 37(15):1734–40. 10.1080/02640414.2019.1589920 30860956

[52] Hurst P Ring C Kavussanu M. Athletes using ergogenic and medical sport supplements report more favourable attitudes to doping than non-users. J Sci Med Sport. (2021) 24(3):307–11. 10.1016/j.jsams.2020.09.012 32998850

[53] Hurst P Ring C Kavussanu M. Moral values and moral identity moderate the indirect relationship between sport supplement use and doping use via sport supplement beliefs. J Sports Sci. (2022) 40(10):1160–7. 10.1080/02640414.2022.2053387 35301930

[54] Hurst P. Are dietary supplements a gateway to doping? A retrospective survey of Athletes’ substance use. Subst Use Misuse. (2023) 58(3):365–70. 10.1080/10826084.2022.2161320 36645808

[55] Hurst P Ng PY Under L Fuggle C. Dietary supplement use is related to doping intention via doping attitudes, subjective norms, and perceived behavioural control. Perform Enhanc Health. (2024) 12(2):100278. 10.1016/j.peh.2024.100278

[56] Kristensen JÅ Haugen T Ommundsen Y. Supplement usage and doping attitudes in elite youth sports: the mediating role of dietary supplement acceptance. PLoS One. (2024) 19(2):e0297078. 10.1371/journal.pone.0297078 38300939 PMC10833512

[57] Abreu R Oliveira CB Brito J Teixeira VH. Perspectives and practices of nutritionists on dietary supplements for elite soccer teams: a cross-sectional survey study. Front Sports Act Living. (2023) 5:1–8. 10.3389/fspor.2023.1230969 PMC10450918 37637220

[58] Boardley ID Grix J Ntoumanis N Smith AL. A qualitative investigation of coaches’ doping confrontation efficacy beliefs. Psychol Sport Exerc. (2019) 45:101576. 10.1016/j.psychsport.2019.101576

[59] Engelberg T Moston S. Inside the locker room: a qualitative study of coaches’ anti-doping knowledge, beliefs and attitudes. Sport Soc. (2016) 19(7):942–56. 10.1080/17430437.2015.1096244

[60] Fruchart E Rulence-Pâques P Mullet E. Mapping adults’ and young athletes’ views regarding how acceptable it is to use a nutritional supplement in sport. Int J Sport Exerc Psychol. (2019) 17(5):477–92. 10.1080/1612197x.2017.1367952

[61] Patterson LB Backhouse SH Duffy PJ. Anti-doping education for coaches: qualitative insights from national and international sporting and anti-doping organisations. Sport Manag Rev. (2016) 19(1):35–47. 10.1016/j.smr.2015.12.002

[62] Haubenstricker JE Lee JW Segovia-Siapco G Medina E. The theory of planned behaviour and dietary behaviours in competitive women bodybuilders. BMC Public Health. (2023) 23(1):1–14. 10.1186/s12889-023-16568-w 37667272 PMC10476312

[63] Königstein K Gatterer K Weber K Schmidt-Trucksäss A Tercier S Blank C. Geographical heterogeneity of doping-related knowledge, beliefs and attitude among 533 youth olympics participants. J Sci Med Sport. (2021) 24(11):1116–22. 10.1016/j.jsams.2021.06.001 34176766

[64] Kuff RF Lucchese-Cheung T Quevedo-Silva F Giordani AM. Building muscles from eating insects. Sustainability. (2023) 15(22):15946. 10.3390/su152215946

[65] Langan-Evans C Cronin C Hearris MA Elliott-Sale KJ Morton JP. Perceptions of current issues in female sport nutrition from elite athletes, practitioners, and researchers. Women Sport Phys Activity J. (2022) 30(2):133–43. 10.1123/wspaj.2022-0004

[66] Muwonge H Zavuga R Kabenge PA. Doping knowledge, attitudes, and practices of Ugandan athletes’: a cross-sectional study. Subst Abuse Treat Prev Policy. (2015) 10:1. 10.1186/s13011-015-0033-2 26395767 PMC4579610

[67] Placentino U Sogari G Viscecchia R De Devitiis B Monacis L. The new challenge of sports nutrition: accepting insect food as dietary supplements in professional athletes. Foods. (2021) 10(5):1117. 10.3390/foods10051117 34070020 PMC8157859

[68] Sak D Dayi T Günay E Öni˙Z A. Nutritional knowledge and ergogenic aid using Status of competitive and recreational cyclists. Pamukkale J Sport Sci. (2022) 13(3):131–45. 10.54141/psbd.1143549

[69] Turfus SC Smith JOL Mansingh A Alexander-Lindo RL Roopchand-Martin S. Supplementation practices, perceptions and knowledge about anti-doping among Jamaican high school athletes. Perform Enhanc Health. (2019) 7(1-2):100145. 10.1016/j.peh.2019.07.001

[70] Wardenaar FC Hoogervorst D Vento KA De Hon O. Dutch Olympic and non-Olympic athletes differ in knowledge of and attitudes toward third-party supplement testing. J Diet Suppl. (2021) 18(6):646–54. 10.1080/19390211.2020.1829248 33021113

[71] Wirnitzer K Motevalli M Tanous DR Gregori M Wirnitzer G Leitzmann C Sex differences in supplement intake in recreational endurance runners—results from the NURMI study (step 2). Nutrients. (2021) 13(8):2776. 10.3390/nu13082776 34444935 PMC8402241

[72] Yasuda J Myoenzono K Takai E Toguchi M Tsunezumi S Kondo C Importance of “meal first” strategy and effective situations of supplement use in elite athletes: Japan high performance sport center position stand. Front Sports Act Living. (2023) 5:1–9. 10.3389/fspor.2023.1188224 PMC10293618 37383062

[73] Burke LM. Practical issues in evidence-based use of performance supplements: supplement interactions, repeated use and individual responses. Sports Med. (2017) 47(S1):79–100. 10.1007/s40279-017-0687-1 28332111 PMC5371635

[74] Close GL Kasper AM Walsh NP Maughan RJ. “Food first but not always food only”: recommendations for using dietary supplements in sport. Int J Sport Nutr Exerc Metab. (2022) 32(5):371–86. 10.1123/ijsnem.2021-0335 35279015

[75] Edenfield KM. Sports supplements. Primary Care: Clinics in Office Practice. (2020) 47(1):37–48. 10.1016/j.pop.2019.10.002 32014135

[76] Garthe I Maughan RJ. Athletes and supplements: prevalence and perspectives. Int J Sport Nutr Exerc Metab. (2018) 28(2):126–38. 10.1123/ijsnem.2017-0429 29580114

[77] Maughan RJ Depiesse F Geyer H. The use of dietary supplements by athletes. J Sports Sci. (2007) 25(sup1):S103–13. 10.1080/02640410701607395 18049988

[78] Molinero O Márquez S. (s. f.). Use of nutritional supplements in sports: Risks, knowledge, and behavioural-related factors. 19593480

[79] Barbany Cairó JR. Innovación y nuevas perspectivas en la alimentación para el deporte. Ayudas ergogénicas en desarrollo: prebióticos, calostro, óxidos de nitrógeno y otras. En A. Lizarraga Dallo, J. R. Barbany Cairó, V. Pons Salas, E. Pasabán Lizarribar, & L. Capdevila Auguets, Alimentación y deporte: tendencias actuales, tecnología, innovación y pedagogía (pp. 29-40). Instituto Tomás Pascual Sanz para la Nutrición y la Salud (2010).

[80] De Oliveira GT De Souza HLR Meireles A Dos Santos MP Leite LHR Ferreira RM Use of ergogenic aids among Brazilian athletes: a cross-sectional study exploring competitive level, sex and sports. Front Sports Act Living. (2023) 5:1–7. 10.3389/fspor.2023.1257007 PMC10556477 37808161

[81] Castillo Díaz P Cabrera Oliva V Ramírez-Reyes L. Los suplementos nutricionales en el deporte de alto rendimiento y proyectos de su desarrollo futuro en Cuba. Revista Peruana de Ciencia de la Actividad Física y del Deporte. (2023) 10(1):1590–604. Available online at: https://www.rpcafd.com/index.php/rpcafd/article/view/241

[82] Jowett GE Stanger N Madigan DJ Patterson LB Backhouse SH. Perfectionism and doping willingness in athletes: the mediating role of moral disengagement. Psychol Sport Exerc. (2023) 66:102402. 10.1016/j.psychsport.2023.102402 37665864

[83] Boardley ID Smith AL Ntoumanis N Gucciardi DF Harris TS. Perceptions of coach doping confrontation efficacy and athlete susceptibility to intentional and inadvertent doping. Scand J Med Sci Sports. (2019) 29(10):1647–54. 10.1111/sms.13489 31148275

[84] Connor JM. Towards a sociology of drugs in sport. Sport Soc. (2009) 12(3):327–8. 10.1080/17430430802673676

[85] Cook GM Fletcher D Carroll C. Psychosocial functioning of Olympic coaches and its perceived effect on athlete performance: a systematic review. Int Rev Sport Exerc Psychol. (2020) 14(1):1–34. 10.1080/1750984X.2020.1802769

[86] Antuñano NPG Marqueta PM Redondo RB Fernández CC Bonafonte LF Aurrekoetxea TG Suplementos nutricionales para el deportista: Ayudas ergogénicas en el deporte-2019. Documento de consenso de la Sociedad Española de Medicina del Deporte. Archivos de Medicina del Deporte. (2019) 36(Suppl S1):7–83.

[87] Medrano-Echeverría M Cadenas-Sánchez C Alfaro-Magallanes VM Labayen Goñi I. Nutrición deportiva. En F. C. Ibáñez Moya & M. J. Beriain Apesteguía (Coords.). Nutrición y dietética: De la teoría a la práctica (2^a^ ed.). Ediciones Eunate (2024). p. 333–50.

